# Kinetically inert manganese (II)-based hybrid micellar complexes for magnetic resonance imaging of lymph node metastasis

**DOI:** 10.1093/rb/rbad053

**Published:** 2023-05-25

**Authors:** Kai Chen, Zhongyuan Cai, Yingzi Cao, Lingling Jiang, Yuting Jiang, Haojie Gu, Shengxiang Fu, Chunchao Xia, Su Lui, Qiyong Gong, Bin Song, Hua Ai

**Affiliations:** National Engineering Research Center for Biomaterials, Sichuan University, Chengdu 610064, China; National Engineering Research Center for Biomaterials, Sichuan University, Chengdu 610064, China; National Engineering Research Center for Biomaterials, Sichuan University, Chengdu 610064, China; National Engineering Research Center for Biomaterials, Sichuan University, Chengdu 610064, China; National Engineering Research Center for Biomaterials, Sichuan University, Chengdu 610064, China; National Engineering Research Center for Biomaterials, Sichuan University, Chengdu 610064, China; National Engineering Research Center for Biomaterials, Sichuan University, Chengdu 610064, China; Department of Radiology, West China Hospital, Sichuan University, Chengdu 610041, China; Department of Radiology, West China Hospital, Sichuan University, Chengdu 610041, China; Huaxi MR Research Center (HMRRC), Department of Radiology, Frontiers Science Center for Disease-Related Molecular Network, State Key Laboratory of Biotherapy, West China Hospital, Sichuan University, Chengdu 610041, China; Functional and Molecular Imaging Key Laboratory of Sichuan Province, Key Laboratory of Transplant Engineering and Immunology, NHC, Research Unit of Psychoradiology, Chinese Academy of Medical Sciences, Chengdu 610041, China; Department of Radiology, West China Xiamen Hospital of Sichuan University, Fujian, Xiamen 361000, China; Department of Radiology, West China Hospital, Sichuan University, Chengdu 610041, China; Department of Radiology, Sanya People’s Hospital, Sanya 572000, China; National Engineering Research Center for Biomaterials, Sichuan University, Chengdu 610064, China; Department of Radiology, West China Hospital, Sichuan University, Chengdu 610041, China

**Keywords:** kinetically inert, amphiphilic manganese chelate, T1 contrast agent, sentinel lymph node, magnetic resonance imaging

## Abstract

The localization and differential diagnosis of the sentinel lymph nodes (SLNs) are particularly important for tumor staging, surgical planning and prognosis. In this work, kinetically inert manganese (II)-based hybrid micellar complexes (MnCs) for magnetic resonance imaging (MRI) were developed using an amphiphilic manganese-based chelate (C18-PhDTA-Mn) with reliable kinetic stability and self-assembled with a series of amphiphilic PEG-C18 polymers of different molecular weights (C18En, *n* = 10, 20, 50). Among them, the probes composed by 1:10 mass ratio of manganese chelate/C18En had slightly different hydrodynamic particle sizes with similar surface charges as well as considerable relaxivities (∼13 mM^−1^ s^−1^ at 1.5 T). *In vivo* lymph node imaging in mice revealed that the MnC MnC-20 formed by C18E20 with C18-PhDTA-Mn at a hydrodynamic particle size of 5.5 nm had significant signal intensity brightening effect and shortened *T*_1_ relaxation time. At an imaging probe dosage of 125 μg Mn/kg, lymph nodes still had significant signal enhancement in 2 h, while there is no obvious signal intensity alteration in non-lymphoid regions. In 4T1 tumor metastatic mice model, SLNs showed less signal enhancement and smaller *T*_1_ relaxation time variation at 30 min post-injection, when compared with normal lymph nodes. This was favorable to differentiate normal lymph nodes from SLN under a 3.0-T clinical MRI scanner. In conclusion, the strategy of developing manganese-based MR nanoprobes was useful in lymph node imaging.

## Graphical abstract

**Figure:**
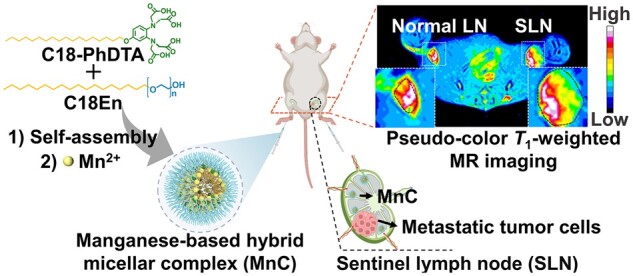

## Introduction

Among various routes of tumor metastasis, lymphogenous metastasis is a common way, and the first lymph nodes (LNs) invaded by tumor cells are the sentinel lymph nodes (SLNs) [1–3]. In breast cancer patients, the localization and diagnosis of SLNs are important to surgical planning and prognosis [4]. In current clinical practice, localization of the SLNs during surgery by means of dyes (such as methylene blue, iso-sulfan blue and Patent Blue V^®^) or radioactive labeling tracers (such as technetium 99 sulfur colloids and Lymphoseek^®^) and intraoperative biopsy to differ LN metastasis, which increases the complexity of the procedure and leads to prolonged operative time and may result in surgical complications [2]. In addition, dyes have weak optical penetration, tend to contaminate normal tissues and cause allergy-like reactions, while radiocolloids have poor spatial resolution and increase radiation exposure for both surgeons and patients [5, 6]. Indocyanine green (ICG)-guided LN localization is active in clinical trials, but the need to use special imaging devices for visualization of ICG during surgery leads to higher costs [7–9].

Preoperative diagnostic staging of LNs to determine the surgical planning in advance is a more desired approach [10–12]. Medical imaging techniques have been developed for clinical diagnosis and biomaterials evaluation [13]. Magnetic resonance imaging (MRI) is a promising method for preoperative LN staging, because of its higher contrast of soft tissue than computed tomography, absence of radiation and superior resolution and depth of penetration than fluorescence imaging. However, the lack of sensitivity has limited MRI to stage LNs, and the use of contrast agents could help to improve this ability [14, 15].

In the last two decades, a variety of magnetic resonance (MR) contrast agents have been developed and explored for their use in LN imaging [4, 16–18]. Nanoparticles have an inherent ability to localize LNs due to their property of accumulating in LNs with the flow of lymph fluid [19–22]. For example, superparamagnetic iron oxide nanoparticles were expected to be used to address preoperative staging of LNs due to their good biosafety and excellent *T*_2_ relaxivities [23–25]. Combidex^®^ was explored in clinical trials, but its magnetic artifacts and lack of accumulation in LNs via intravenous injection may hinder their further application [23, 26, 27]. Gadomer-17^®^, a dendritic macromolecular gadolinium chelate, is a contrast agent designed for vascular imaging. With a suitable particle size for rapid accumulation in LNs and efficient clearance by the kidney, Gadomer-17^®^ has been tested for LN imaging [22, 24, 28, 29]. However, although clinical trials have been reported, further development of Gadomer-17^®^ was abandoned, possibly due to concerns about the safety of gadolinium ion and financial hurdles of development costs [30–32].

Manganese, an essential trace element in our body, is a safer alternative to gadolinium ion that can be used for *T*_1_ imaging. In recent years, the development of kinetically inert ligands has opened up the possibility of clinical applications of manganese-based contrast agents [33–37]. For example, manganese complexes formed by the ethylenediaminetetraacetic acid (EDTA)-analog o-phenylenediamine-N,N,N′,N′-tetraacetic acid (PhDTA) were found to be highly kinetically stable with a dissociation half-life of up to 19 h [38]. The findings of this type of manganese ligand offer the possibility of developing kinetically inert manganese chelate-based nanoparticles.

**Scheme 1.**
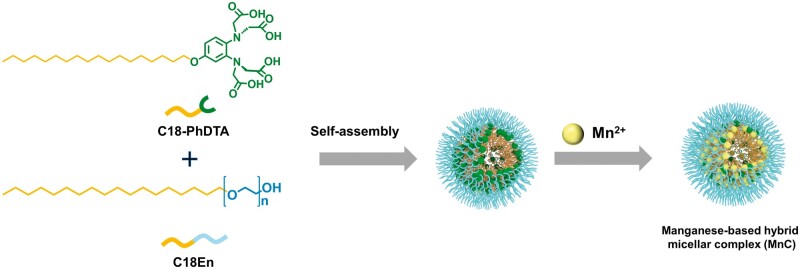
Schematic illustration of C18-PhDTA and polyethylene glycol monooctadecyl ether (C18En) assembled into mixed micelles, and further chelated with manganese to form manganese-based hybrid micellar complexes.

In this work, we synthesized the amphiphilic ligand C18-PhDTA via the alkylation modification of PhDTA, which could further form homogeneous micelles by self-assembly with amphiphilic polyethylene glycol (PEG)-based polymers, such as PEG monooctadecyl ether, and Pluronic F-127 (Scheme 1). After chelated with manganese ions, the as-prepared manganese-based hybrid micellar complexes (MnCs) were characterized by dynamic light scattering (DLS), matrix-assisted laser desorption ionization time-of-flight mass spectrometry (MALDI-TOF-MS), and transmission electron microscope (TEM). The relaxivities, transmetalation kinetics, biosafety and LN imaging capability with different dosages were also investigated. Finally, models of SLN metastasis from breast cancer in mice were established, and the MnCs were tested for differential diagnosis of LN metastasis. This work can provide some insights for the design of manganese-based hybrid micellar contrast agents for imaging of LN metastasis.

## Materials and methods

### Materials

4-amino-3-nitrophenol, 1-iodooctadecane, Pluronic F127 (M_W_ ∼ 12 600 g·mol^−1^) and a series of PEG monooctadecyl ether (C18En, *n* = 10, 20, 50) were purchased from TCI (Shanghai) Chemical Industry Development Corporation. Potassium tert-butylate (KO*t*Bu), ethyl bromoacetate, N,N-diisopropylethylamine (DIPEA) and stannous chloride dihydrate were purchased from Aladdin (Shanghai) Biochemical Technology Co., Ltd. Anhydrous N,N-dimethylformamide (DMF) and anhydrous acetonitrile were purchased from J&K (Beijing) Scientific Co., Ltd. Potassium iodide, hydrochloric acid, ethanol, ammonium chloride and other reagents were purchased from Kelong (Chengdu) Chemical Co., Ltd. All reagents and solvents were used directly without further purification.

### Octadecyloxy modification of ortho-phenylenediamine-N,N,N′,N′-tetraacetic acid (C18-PhDTA)

The synthesis route was modified from previous literatures [39]. The synthesis flow chart is shown in Supplementary Scheme S1.

#### Preparation of 2-nitro-4-(octadecyloxy)aniline (2)

4-Hydroxy-2-nitroaniline (4.00 g, 25.95 mmol), 30 ml DMF with t-BuOK (3.20 g, 28.25 mmol) was added to a round bottom flask. Kept at 0°C for 30 min, iodooctadecane (10.86 g, 28.55 mmol) was added dropwise under nitrogen flow, then the reaction mixture was maintained at 0°C for 3 h. The reaction was monitored by thin-layer chromatography (TLC), and the mixture was quenched by adding 500 ml of aqueous NH_4_Cl solution after reaction. The solid was collected by filtration and rinsed with deionized water until the filtrate became colorless, and the filter cake was dried under vacuum to give a bright red paraffin-like solid 2 in 56% yield. 1H NMR (400 MHz, Chloroform-d, δ) 7.53 (1H, d, NCCH aromatic), 7.06 (1H, d, OCCH aromatic), 6.76 (1H, s, CH aromatic), 3.98–4.03 (2H, t, OCH2), 1.78 (2H, m, OCH2**CH2**), 1.56 (2H, m, OCH2CH2**CH2**), 1.41 (2H, m, OCH2CH2CH2**CH2**), 1.26 (26H, m, (CH2)13), 0.88 (2H, t, CH3); MS (ESI+): m/z calcd for C24H42N2O3: 406.32, found: 407.33 (M + H) + 100%.

#### Preparation of tetraethyl 2,2′,2″,2′″-((4-(octadecyloxy)-1,2-phenylene)bis(azanet-riyl))tetraacetate) (3)

Two (2.23 g, 5.48 mmol), stannous chloride dihydrate (5.07 g, 21.92 mmol), deionized water (1 ml) acetonitrile (30 ml) and anhydrous ethanol (30 ml) were added to a round bottom flask. Monitored by TLC, the reaction refluxed for 3 h under nitrogen gas. After the reaction, the mixture was quenched by adding 150 ml of saturated sodium carbonate and then extracted with dichloromethane (100 ml × 5). The dichloromethane phases were combined, dried overnight using anhydrous sodium sulfate, and then the solute is collected. Ethyl bromoacetate (6.70 ml, 10.1 g), DIPEA (8.30 ml, 6.16 g), sodium iodide (1.30 g, 8.67 mmol), acetonitrile (10 ml) and the intermediate were added to a round bottom flask and refluxed for 7 h. Then, after cooling to room temperature and the solvent was removed. Water (50 ml) was added to dissolve the residue and then extracted using chloroform (3 × 40 ml). The chloroform phases were combined, dried overnight using anhydrous sodium sulfate, and then dried under reduced pressure to obtain a brown oil. Purification was performed with flash silica gel chromatography using petroleum ether: ethyl acetate = 10:1 to obtain a colorless oil liquid in 60% yield. 1H NMR (600 MHz, Chloroform-d, δ) 6.97 (1H, d, CH aromatic), 6.46–6.58 (2H, m, CH aromatic), 4.20–4.31 (8H, s, NCH2), 4.11 (8H, m, COOCH2), 3.85 (2H, t, O**CH2**), 1.72 (2H, m, OCH2**CH2**), 1.42 (2H, m, OCH2CH2**CH2**), 1.26 (28H, m, (CH2)14), 1.20 (12H, t, COOCH2**CH3**), 0.87 (3H, t, (CH2)14**CH3**); MS (ESI+): m/z calcd for C40H68N2O9: 720.49, found: 721.5 (M + H) + 100%.

#### Preparation of C18-PhDTA

Three (4.62 g, 6.41 mmol) were dissolved in 40 ml of ethanol and NaOH (1.28 g, 32 mmol) was dissolved in 5 ml of deionized water, and then the base was added dropwise to the ethanol solution under stirring. After heating and refluxing for 18 h, the light yellow precipitate was formed. The precipitate was filtered and then dissolved into water. The pH was adjusted to 2 using concentrated hydrochloric acid to produce the precipitate, which was separated by filtration and washed with ice water (3 × 5 ml). The sample was dried to constant weight to obtain an off-white solid in 80% yield. 1H NMR (600 MHz, DMSO-*d6*, δ) 6.68 (1H, s, NC**CH**CH aromatic), 6.41 (2H, m, OC**CH** aromatic), 4.10–4.23 (8H, s, NCH2), 3.82 (2H, t, O**CH2**), 1.66 (2H, q, OCH2**CH2**), 1.38 (2H, q, OCH2CH2**CH2**), 1.24 (28H, m, (CH2)14), 0.85 (3H, t, (CH2)14**CH3**); MS (ESI−): m/z calcd for C32H48N2O9: 604.37, found: 607.38 (M + 3H) – 100%.

#### Preparation of C18-PhDTA-Mn

C18-PhDTA (1.00 g 1.64 mmol) was dissolved in 20 ml of Tris buffer (pH = 7.4) and MnCl_2_·4H_2_O (0.36 g, 1.81 mmol) was dissolved in 2 ml of Tris buffer, respectively. MnCl_2_ solution was added dropwise to the C18-PhDTA solution under the protection of nitrogen. The pH was then adjusted to 7.4 using 1 M aqueous sodium hydroxide. Subsequently, dialysis was performed for three days to remove Tris and excess manganese ions. Finally, the product was concentrated and lyophilized to obtain C18-PhDTA-Mn. MS (ESI−): m/z calcd for C32H48MnN2O9: 659.68, found: 660.30 (M + H)−, 682.30 (M + Na)−.

### Preparation of hybrid micellar complexes

The hybrid micelles were prepared by co-assembly of amphiphilic PEG polymer with C18-PhDTA followed by chelation of Mn (II) ions. The amphiphilic PEG polymer used in this work included Pluronic F127(M_W_ ∼ 12 600 g·mol^−1^) and a series of PEG monooctadecyl ether (C18En, *n* = 10, 20, 50). Briefly, 1 equivalent of C18-PhDTA was dissolved in DMSO, and then amphiphilic PEG polymer was added in a mass ratio (C18-PhDTA/polymer = 1:1, 1:3, 1:5, 1:8 and 1:10). The DMSO solution was then added dropwise to an aqueous sodium hydroxide solution at pH = 8 under ultrasonication, and then the pH of the mixture was readjusted to 7–8 using 1 M aqueous sodium hydroxide. Subsequently, 1.2 equivalents of aqueous manganese chloride solution were added under nitrogen protection, and the pH was readjusted to 7–8 again. Then dialysis was performed for 3 days to remove the excess manganese and sodium ions. The product solution was concentrated and the manganese content was determined by inductively coupled plasma optical emission Spectrometer (ICP-OES, Agilent 5100 SVDV).

### Structure and morphology characterization of hybrid micellar complexes

1H NMR (400 MHz) spectra of C18-PhDTA were recorded on an Ultra Shield 600 spectrometer (Bruker AV II-600 MHz) in DMSO-*d6*. The molecular weight of C18-PhDTA and C18-PhDTA-Mn were checked by a Liquid Chromatography–Electrospray Ionization–Mass Spectrometry (LC-ESI-MS, Bruker impact II). TEM (JEOL JEM-2100 Plus) was used to observe the morphology of the hybridized micelles. The hydrodynamic particle size as well as the surface potential before and after chelation of manganese ions were determined by Zeta Sizer (Nano-ZS90, Malvern Instruments, Ltd, UK). After lyophilization of the hybrid micelles, the percentage of manganese content was estimated using energy-dispersive spectrometer (EDS) on Hitachi S-4800 scanning electron microscope (Japan) and the chelation of manganese ions in the micelles was determined by MALDI-TOF-MS (Bruker AutoFlex III). Among the hybrid micelles, the MnCs formed by the self-assembly of C18-PhDTA-Mn with C18E10, C18E20, C18E50 and F127 according to the mass ratio of 1:10 were named MnC-10 MnC-20, MnC-50 and MnC-127, respectively.

### Critical micelle concentration measurement

The pyrene fluorescence method was used to determine the critical micelle concentration (CMC) of MnC-10 MnC-20, MnC-50 and MnC-127. Briefly, the solid content of the sample was determined by lyophilization, and then the sample was prepared as a gradient solution from 1.25 × 10^−5^ to 0.75 mg/ml, for a total of 18 concentrations. A preconfigured acetone solution of pyrene (6.1 mol/l) was added to the sample glass bottle (100 μl/bottle), and the solution of the sample (4 ml) was added after the acetone evaporated and then mixed well. The ratio of fluorescence intensity at 338 and 334 nm (I_338_/I_334_) was subsequently measured using an F-7000 fluorescence spectrophotometer at an excitation wavelength of 395 nm. I_338_/I_334_ versus the concentrations were plotted, and the concentration corresponding to the mutation point was the CMC.

### Transmetalation kinetics

Transmetalation kinetics of MnC-10 MnC-20, MnC-50 and MnC-127 were verified according to previous literatures [40, 41]. Proton relaxation rates of water molecules containing 0.1 mmol Mn/l of manganese hybridized micelles were determined over 48 h under several conditions. Among them, 0.15, 0.3 and 0.6 mmol/l of zinc ion or EDTA were used for competitive ligand experiments with pH maintained at 7.4 using tris(hydroxymethyl)aminomethane (Tris) buffer. Acetate buffer with pH = 5.0 was used instead of Tris buffer for the experiments under acidic conditions. Relaxation rate measurements were performed on a Siemens Skyra 3.0-T MR scanner with the spin echo (SE) sequences.

### *T*_1_ and *T*_2_ relaxivity measurement *in vitro*

In this experiment, the hybrid micelles were configured into solutions with different manganese ion concentrations (0.5, 0.4, 0.3, 0.25, 0.15, 0.1, 0.06, 0.03 mM). Then, *T*_1_-weighted images, *T*_2_-weighted images and relaxation times were obtained on a 1.5-T (μMR 588, UIH, PRC) and 3.0-T (Signa Architect, GE Medical, USA) clinical MRI scanner at room temperature using SE sequences. The reciprocal of the relaxation time is the relaxation rate, which is linearly fitted versus the concentration, and the slope of the line obtained is the relaxivity. The parameters used as follow: *T*_1_-weighted images at 1.5 T (echo time (TE) = 13.9 ms, repetition time (TR) = 90 ms, slice thickness = 3.7 mm, field of view (FoV) = 180 × 120 mm, flip angle (FA) = 90°); *T*_2_-weighted images at 1.5 T (TE = 75 ms, TR = 3500 ms, slice thickness = 3.7 mm, FoV = 180 × 120 mm, FA = 90°).

### Cytotoxicity studies

The cytotoxicity of MnC-10, MnC-20, MnC-50 and MnC-127 to Raw264.7 was evaluated by 3-(4,5)-dimethylthiahiazo (-z-y1)-3,5-di- phenytetrazoliumromide (MTT) assay. Raw264.7 cells were inoculated in 96-well plates. After 24 h of culture, different concentrations of hybrid micelles were added and incubated for the other 12 h. After the well was washed using phosphate buffered saline (PBS) for three times, 0.5% MTT solution was added to each well and incubated for another 4 h. After 200 μl DMSO was added, the absorbance was measured at 490 nm, and the cell viability was calculated according to the previous literatures [40].

### Histological and toxicological evaluation *in vivo*

Fifteen BALB/c mice (18–22 g) were randomly divided into five groups (*n* = 3). Then, four groups were administered subcutaneously on both hindfoot at a dosage of 500 μg Mn/kg body weight (BW) with the four MnCs, respectively, and the other group was treated with saline of same volume. After 24 h, the blood samples were collected, and the serum was obtained by centrifugation at 4°C with 3000 rpm for 30 min. Then, the biochemical indicators of liver function (alanine transferase (reference range: 10.06–96.47), aspartate transferase (reference range: 36.31–235.48), total bilirubin (reference range: 6.09–53.06), albumin (reference range: 21.22–39.15) and alkaline phosphatase (reference range: 22.52–474.35)) and kidney function (the blood urea nitrogen (reference range: 10.81–34.74), creatinine (reference range: 10.91–85.09), serum total protein (reference range: 38.02–75.06), cholesterol (reference range: 2.05–4.16) and the blood glucose (reference range: 4.66–13.42)) were evaluated by serum biochemical analysis. The mice were sacrificed with excess isoflurane, and major organs including the heart, liver, spleen, lung and kidney were taken out, washed with PBS and treated for histological analysis after fixed with 4% paraformaldehyde.

### Animal experiment

All animal experiments were conducted in accordance with the standard of the Biomedical Research Ethics Committee of West China Hospital of Sichuan University. Female balb/c mice were purchased from Dossy (Chengdu) Experimental Co. All images were acquired by *T*_1_-weighted sequences from a siemens 3.0-T skyra MRI scanner equipped with a mouse coil with the following parameters: TE = 13 ms, TR = 500 ms, slice thickness = 1.5 mm, FA = 150°, FoV = 35 mm × 35 mm, acquisition matrix = 192 mm × 192 mm, number of averages = 6.

### Comparison of different MnC formulations *in vivo* MR images

After mice were anesthetized using 2% isoflurane gas flow, MnCs with different polymers were injected subcutaneously into the hindfeet of healthy mice at 1000 μg/kg BW. Three of the mice were injected with MnC-10 in the right hindfoot and MnC-20 in the left hindfoot, while the other three mice were injected with MnC-127 in the left hindfoot and MnC-50 in the right hindfoot. *T*_1_-weighted images and *T*_1_-mapping pseudo-color images of popliteal LNs were acquired before, 30, 60 and 120 min after injection.

### *In vivo* MR imaging of lymph node of MnC-20 with different dosages

Anesthetized using 2% isoflurane gas flow, mice were injected subcutaneously in the hindfeet with different dosages of MnC-20. A total of six mice were used, three of which were injected with 750 μg Mn/kg BW in the right hindfoot, while the left hindfoot was administered with 500 μg Mn/kg BW, and the other three were injected with 250 μg Mn/kg BW in the right hindfoot, while the left foot was administered with 125 μg Mn/kg BW. *T*_1_-weighted images and *T*_1_-mapping pseudo-color images of the popliteal LNs were obtained before, 30, 60, and 120 min after injection.

### Lymph node metastasis model of 4T1 tumor

The tumor LN metastasis model was established by subcutaneous injection of 4T1 cells (5 × 10^5^ cells in PBS) on the left hind footpad of Balb/c mice [4, 42]. Half a month later, the enlargement of LNs was observed by MRI. And after the contrast-enhanced imaging, the mice were sacrificed with anesthesia, and pathological sections of the LNs stained with hematoxylin and eosin (H&E) were obtained for to determine the establishment of LN metastasis models.

### *In vivo* MR imaging of sentinel lymph node in metastatic mice

After anesthetizing metastatic mice, 250 μg Mn/kg MnC-20 was injected subcutaneously in both the right and left hindfeet of the mice (*n* = 4). *T*_1_-weighted images and *T*_1_-mapping pseudo-color images of the mice were acquired before, 30, 60, 120 and 180 min after injection. Quantitative analysis of all images was achieved by ImageJ.

## Results and discussion

### Synthesis and characterization of amphiphilic C18-PhDTA and C18-PhDTA-Mn

C18-PhDTA was prepared according to the synthetic routes reported in the literature with slight modifications (Supplementary Scheme S1) [38, 39]. The intermediate products as well as the final ligand C18-PhDTA were confirmed by 1H NMR and ESI-MS spectra (Supplementary Figs S1–S3). Due to the amphiphilic structure, the C18-PhDTA was well dispersed in aqueous solution at pH = 7.4, allowing C18-PhDTA to further chelate manganese ions directly to form C18-PhDTA-Mn complexes. ESI-MS spectra showed the successful preparation of C18-PhDTA-Mn (Supplementary Fig. S4). However, the lyophilized sample was almost insoluble in common organic solvent except in chloroform/methanol mixture (*v*/*v* = 3:2), resulting in difficulty in self-assembly of the pre-chelates with amphiphilic polymers to form stable hybrid nanoparticles. This phenomenon is similar to other previous reports [43, 44]. Therefore, the preparation of Mn-based hybrid MnCs adopted the route of self-assembling C18-PhDTA with amphiphilic polymers first and followed by further chelating manganese ions to improve the colloidal stability.

### Synthesis and characterization of Mn-based hybrid micellar complexes

**Figure 1.**
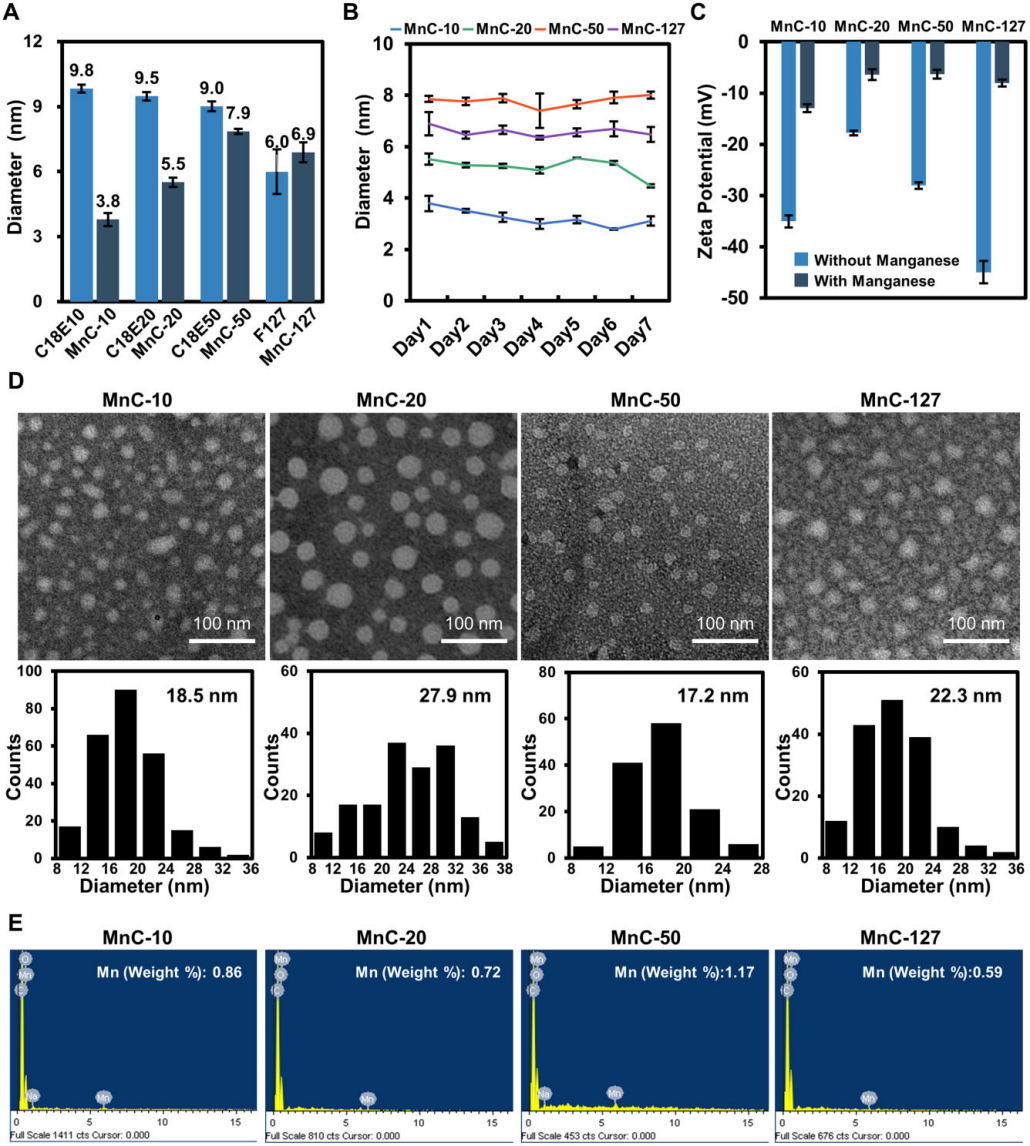
Characterization of manganese-based hybrid micellar complexes (MnCs). (**A**) Hydrodynamic particle diameters of MnC-10, MnC-20, MnC-50, MnC-127 and micelles formed by single type of PEG-based polymers (C18E10, C18E20, C18E50 and F127). (**B**) Size variation of the four MnCs in seven days. (**C**) Zeta potential of the four MnCs before and after chelation with manganese. (**D**) TEM images and particle size distribution calculated by Image J. (**E**) EDS images of the four MnC formulations.

To improve the colloidal stability, PEG-based polymers were introduced, including PEG monooctadecyl ether with different PEG molecular weights (*abbr.* C18E*n*, *n* is the number of EG units, *n* = 10, 20, 50) and Pluronic F127. For each polymer, multiple mass ratios of C18-PhDTA/PEG amphiphilic polymer were used to prepare micelles. As shown in Fig. 1A, Supplementary Fig. S6 and Table S1, all manganese-based hybrid MnCs with C18-PhDTA/polymer mass ratio <1:3 have smaller hydrodynamic particle size compared to the corresponding micelles without C18-PhDTA addition, which may due to the favorable interactions between alkyl groups of C18-PhDTA and C18En, similar to previously reported literature [45–48]. However, larger particle sizes were observed in the 1:1 mass ratio, which may be attributed to the lack of amphiphilic polymers leading to poor colloidal stability [48].

For the hybrid MnCs with mass ratio = 1:10, which exhibited the best colloid stability in all formulations of mass ratios tested by DLS, compositions co-assembled with C18E10, C18E20, C18E50 and F127 were named MnC-10, MnC-20, MnC-50 and MnC-127, respectively. Their surface potentials exhibited a decrease after chelating with manganese ions, indicating chelation process was successful (Fig. 1C). After complexing with manganese ion, hydrodynamic diameters were around 3.8, 5.5, 7.9, and 6.9 nm, respectively, and DLS measurements over 7 days showed that their particle sizes remained almost the same, indicating good colloidal stability (Fig. 1B). The TEM images taken after negative staining with phosphotungstic acid was shown in Fig. 1D, showing that the nanoparticles were spherical, and the average particle size was slightly larger than results measured by DLS, probably due to drying and negative staining in the TEM sample preparation.

In addition, the elemental composition was analyzed by EDS, and the mass content of manganese ions was ∼1%, which is consistent with the feeding ratio: the molecular weight of C18-PhDTA is ∼11 times as large as the molecular weight of manganese ions, and C18-PhDTA is co-assembled with the polymer at a mass ratio of 1:10, leading the calculated content of manganese ions is ∼1% (Fig. 1E). The reliability of approach the assembly followed by chelation was also demonstrated using MALDI-TOF-MS, where mass spectral peaks of C18-PhDTA-Mn could be found, indicating that the manganese ion was indeed chelated to the C18-PhDTA ligand on the MnCs (Supplementary Fig. S5).

The pyrene fluorescence method was used to estimate the CMC to assess the stability of the micelles, which of MnC-10, MnC-20, MnC-50 and MnC-127 is 4.10, 5.29, 14.93 and 84.16 mg/l, while 0.80, 5.36, 7.19 and 191.61 mg/l for the corresponding micelles without C18-PhDTA-Mn, respectively, suggesting that MnC-20 have a similar stability to the one without C18-PhDTA-Mn (Supplementary Figs S7 and S8), while the addition of C18-PhDTA-Mn led to the increased CMC of C18E10 and C18E50 but the decreased CMC of F127.

### Transmetalation kinetics of Mn-based hybrid micellar complexes

**Figure 2.**
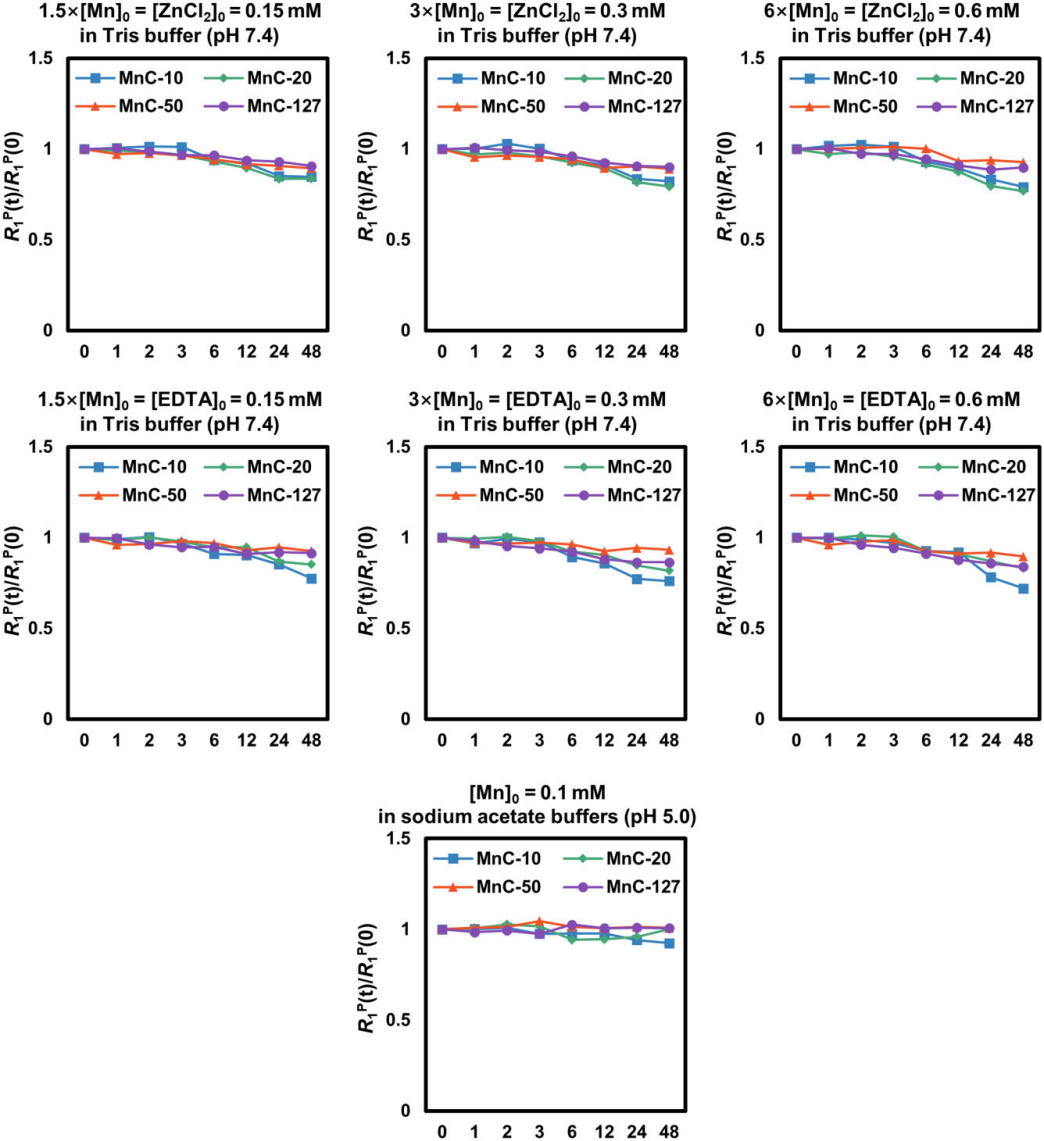
Evolution of longitudinal relaxation rates *R*_1_^p^ (*t*)/*R*_1_^p^ (0) as a function of time for the four MnCs in the presence of Zn^2+^ or EDTA which is 1.5, 3 or 6 times than manganese ion at pH = 7.4, or in the environment of excessive hydrogen protons at pH = 5.0.

Kinetic inertness is particularly important for manganese-based contrast agents [40, 41]. Subsequently, the relaxation rate (*R*_1_^P^(*t*)) method was used to assess the transmetalation kinetics in the exist of divalent metal cations (Zn^2+^), polyaminocarboxylate chelates (EDTA) or under acidic conditions (pH = 5.0 in acetic acid-sodium buffer) (Fig. 2). The concentrations of the competing species (Zn^2+^, EDTA) used were ranged from 1.5 to 6 times the concentration of the manganese complexes. For all concentrations of zinc ions, no significant decrease in *R*_1_^P^(*t*)/*R*_1_^P^(0) was observed in the first 6 h, while a slight decrease was observed only after 12–48 h. This result indicates that even in the presence of a 6-fold concentration of zinc ions, the majority of manganese ions are difficult to be replaced by zinc ions and leak out over a period of several hours, favorable for potential *in vivo* applications. After 24 h, the 6-fold concentration of zinc ions resulted in the most prominent reduction of *R*_1_^P^(*t*)/*R*_1_^P^(0), indicating a concentration-dependent process of zinc ions in replace of manganese ions. EDTA was also used to assess the stability of manganese ion complexes and their competition behavior, and the result was similar to that of zinc ions. Furthermore, no significant decrease in *R*_1_^P^(*t*)/*R*_1_^P^(0) was observed even for up to 48 h in acetic acid-sodium acid buffer at pH = 5.0, indicating a weak competition of hydrogen protons for manganese ions. These experiments demonstrated that the modified PhDTA ligands in micelles were still having a high kinetic inertness similar to that of unmodified PhDTA [38].

### Relaxivity of Mn-based hybrid micellar complexes

**Figure 3.**
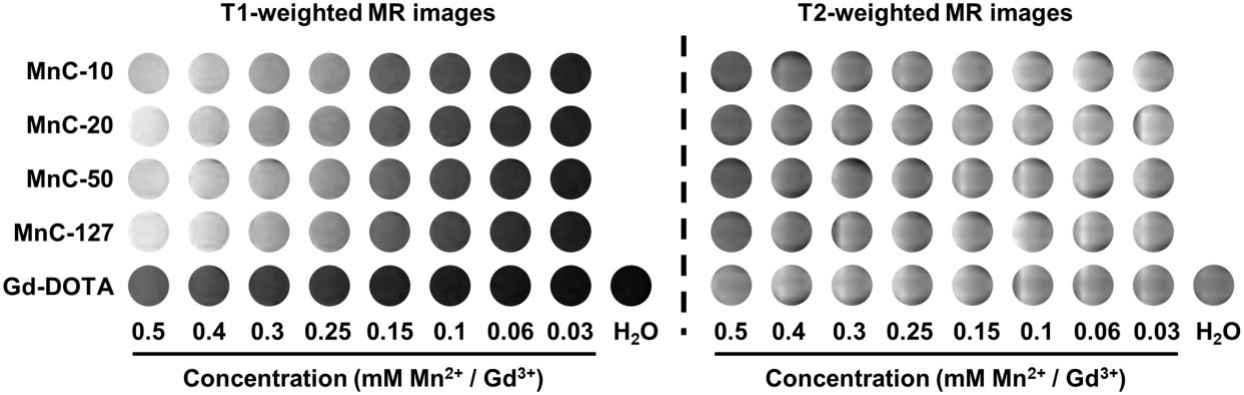
Grayscale *T*_1_-weighted and *T*_2_-weighted MR images at 1.5 T of MnC-10, MnC-20, MnC-50, MnC-127 and the commercial gadolinium-based contrast agent, Gd-DOTA. (TE = 13.9 ms, TR = 90 ms, for *T*_1_-weighted SE sequences and TE = 75 ms, TR = 3500 ms, for *T*_2_-weighted SE sequences).

Relaxivity is a key indicator for evaluating the performance of MR contrast agents *in vitro*. To verify the imaging capability of the manganese-based hybrid MnCs, their signal contrast was measured using the commercial Gd-DOTA as a reference. As shown in Fig. 3 and Supplementary Fig. S12, the four manganese MnCs produced a significant enhancement compared to Gd-DOTA, and the signal intensity at a concentration of 0.15 mM was similar to that of Gd-DOTA at 0.5 mM. Despite different polymers used in the four manganese MnCs, they have similar relaxivities (Table 1). A relaxivity of 13.0 mM^−1^ s^−1^ can be achieved for MnC formulations at 1.5 T, more than three times that of Gd-DOTA (4.0 mM^−1^ s^−1^). Although the relaxivities decreased at 3.0 T, they were still over 7.6 mM^−1^ s^−1^. Notably, in the three MnCs consisting by C18-PhDTA-Mn and C18En, the hydrodynamic particle size increased with the increase of the molecular weight of PEG (Fig. 1A, Supplementary Fig. S13 and Tables S2–S5), and there was a slight increase in the relaxivity, which could be attributed to the increase of the rotational correlation time due to the increase of the nanoparticle size. In addition, the low *r*_2_ of the manganese complexes reflected the stable chelation of manganese ions on the nanocomplexes, as the free manganese ions lead to a higher transverse relaxation time shortening and thus an elevated *r*_2_ [49]. All of the MnCs have an *r*_2_/*r*_1_ that similar to that of Gd-DOTA, indicating the suitability as *T*_1_ contrast agents.

**Table 1.** *T*_1_ and *T*_2_ relaxivities (in concentration of Gd/Mn) of MnC-10, MnC-20, MnC-50, MnC-127 and Gd-DOTA at 1.5 and 3.0 T, respectively

	1.5 T			3.0 T		
	*r*_1_ (mM^−1^ s^−1^)	*r*_2_ (mM^−1^ s^−1^)	*r*_2_/*r*_1_	*r*_1_ (mM^−1^ s^−1^)	*r*_2_ (mM^−1^ s^−1^)	*r*_2_/*r*_1_
MnC-10	13.3	23.8	1.8	8.0	21.7	2.7
MnC-20	13.6	23.8	1.8	7.8	21.3	2.7
MnC-50	13.8	23.8	1.7	8.2	21.8	2.7
MnC-127	13.0	21.8	1.7	7.6	19.8	2.6
Gd-DOTA	4.0	5.2	1.3	3.5	4.0	1.1

### Cytotoxicity studies *in vitro*

Cytotoxicity evaluation was performed as a contrast agent for use in MRI *in vivo*. In this work, Raw264.7 was used to co-incubate with different concentrations of MnCs and cell viability was determined by MTT. The results showed that the MnCs was able to achieve about reliable cell survival at 15 μg/ml and did not have significant cytotoxicity under the lower concentrations (Supplementary Fig. S9). In addition, for the MnC-10, MnC-20 and MnC-50, the increase in molecular weight of PEG improved cell viability and reduced cytotoxicity.

### *In vivo* histological and toxicological evaluation

The biosafety was further assessed by serum biochemical indicators and histological sections (Supplementary Figs S10 and S11). The MnCs were injected unilaterally at a dosage of 500 μg Mn/kg BW in both hindfeet, with the mice injected with same volumes of saline as a control group (*n* = 3). The key serum biochemical indicators were within the normal range. H&E staining of the major organs did not present visible damages. It indicates that the MnCs had good biosafety at this dosage in mice.

### Comparison of MnC formulations *in vivo* MR images

To evaluate the imaging ability of the four contrast agents *in vivo*, the contrast agents were injected subcutaneously in the hindfeet of mice and imaging of popliteal LNs was performed on a clinical 3.0-T MR scanner. Three mice were injected with MnC-10 in the right hindfoot and MnC-20 in the left hindfoot, while the other three mice were injected with MnC-50 in the right hindfoot and MnC-127 in the left hindfoot.

**Figure 4.**
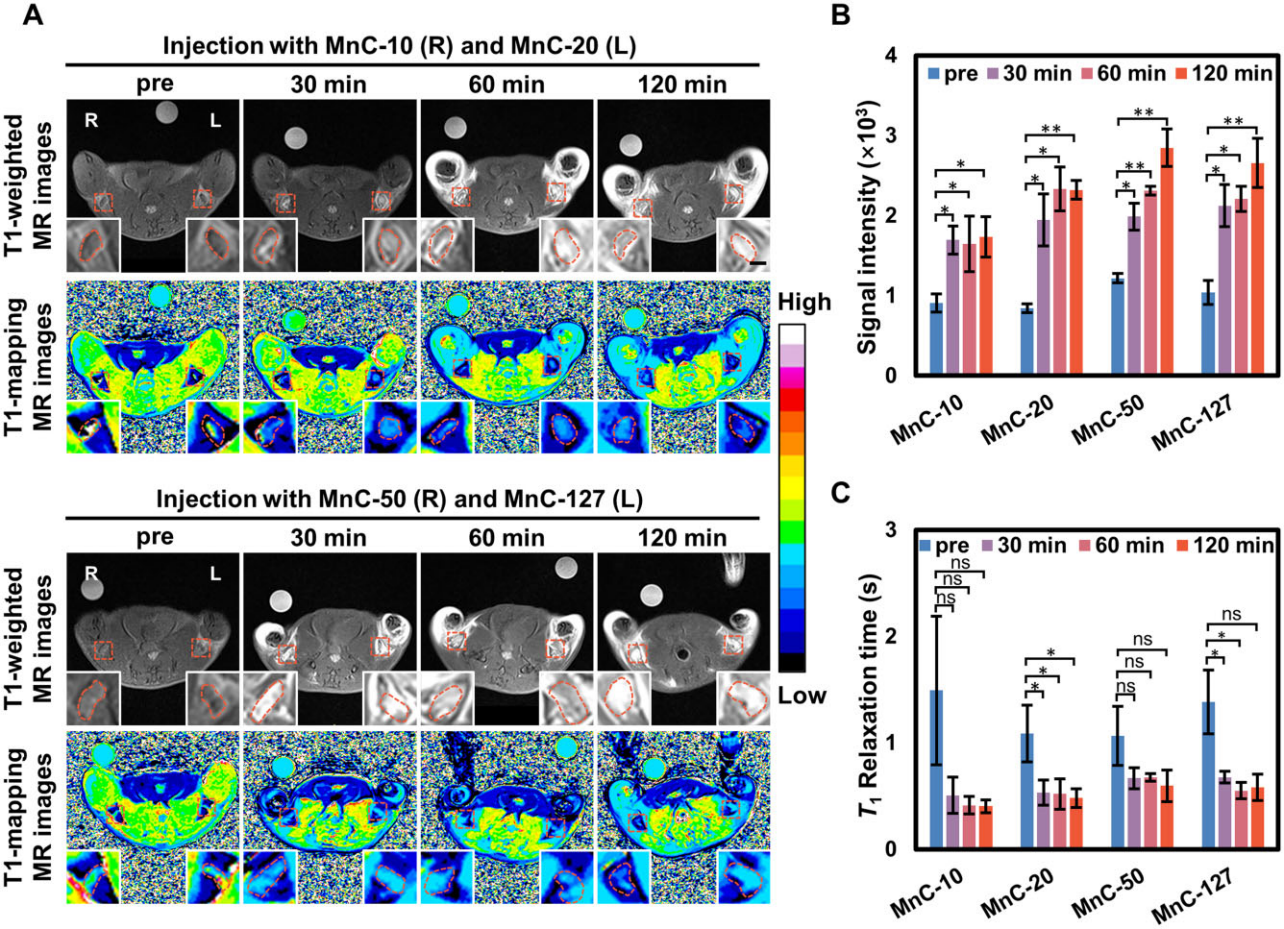
(**A**) *T*_1_-weighted MR images and *T*_1_-mapping pseudo-color images of popliteal lymph nodes before and after injection subcutaneously of MnC-10, MnC-20, MnC-50 and MnC-127 at the dosage of 1000 μg/kg BW, respectively (scalar bar = 1 mm); quantitative analysis of the signal intensity from *T*_1_-weighted MR images (**B**) and *T*_1_ relaxation time from *T*_1_-mapping images (**C**). *P < 0.05; **P < 0.01.

As shown in Fig. 4A, before injection, the LNs appeared as darker images with the signal intensity ranged from 846 to 1219 on the *T*_1_-weighted images, mainly due to their long longitudinal relaxation time (1088–1492 ms). Signal enhancement of the LN was observed at 30 min after probe injection (whose intensity was >1600), and the *T*_1_-mapping pseudo-color images showed the shortening of the relaxation time (drop down to <700 ms). The signal intensity of the *T*_1_-weighted images was measured (Fig. 4B), and all groups showed signal enhancement from 30 to 120 min after injection and revealed significant difference compared with preinjection. Among them, the enhancement caused by MnC-10 was relatively weak and the signal intensity remained almost constant between 30 and 120 min, which may be related to the fact that MnC-10 has the smallest hydrodynamic particle size (∼3.8 nm) with the highest negative surface potential, which may be more easily migrated in the lymphatic system rather than retaining in LNs. From 30 to 60 min post-administration, MnC-20, MnC-50 and MnC-127 groups had similar enhancement. Subsequently, at 120 min, the signal intensity of MnC-20 groups no longer continued to increase, while MnC-50 and MnC-127 groups still went up. In consideration of the similar relaxivity of the four MnCs, this phenomenon suggests that the change in particle size may lead to a variation in the maximum accumulation in the LN, and increased particle size may contribute to the maximum accumulation, resulting in higher contrast.

Similarly, we evaluated the change in *T*_1_ relaxation time. As shown in Fig. 4C, the relaxation times of all groups appeared to be obviously shorter after injection, and their changes between 30 and 120 min were negligible. Among them, there was a statistical difference at all three time points in the group injected with MnC-20 (*P* < 0.05). For the MnC-127 group, the relaxation time has a statistical difference between before and after injection except for 120 min. However, no statistical difference was found for the MnC-10 and MnC-50 groups. Therefore, MnC-20 was selected for further experiments.

### *In vivo* MR images of MnC-20 with different dosages

**Figure 5.**
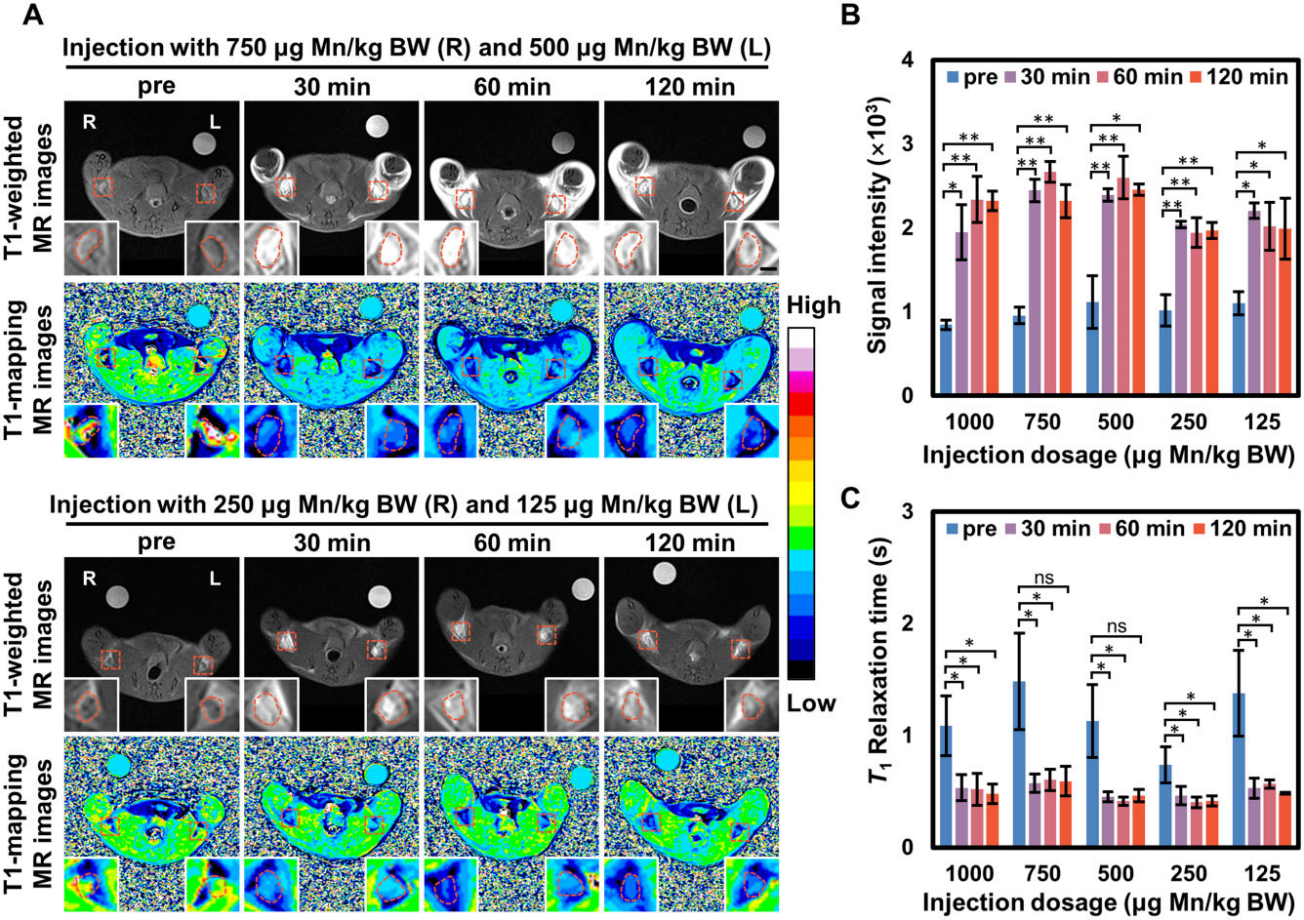
(**A**) *T*_1_-weighted MR images and *T*_1_-mapping pseudo-color images of popliteal lymph nodes before and after injection subcutaneously of MnC-20 at the dosage of 750, 500, 250 and 125 μg/kg BW, respectively (scalar bar = 1 mm); quantitative analysis of the signal intensity from *T*_1_-weighted MR images (**B**) and *T*_1_ relaxation time from *T*_1_-mapping images (**C**), and the data for 1000 μg Mn/kg BW were reproduced from Fig. 4. *P < 0.05; **P < 0.01.

As shown in Fig. 5A, effects of dosages were evaluated in imaging of LNs of MnC-20. A total of six mice with four different dosages were involved, three of which were injected subcutaneously with 750 μg Mn/kg BW of MnC-20 in the right hindfoot and 500 μg Mn/kg BW in the left hindfoot, while the other three were injected with 250 μg Mn/kg BW in the right hindfoot and 125 μg Mn/kg BW in the left hindfoot. *T*_1_-weighted and *T*_1_-mapping images of all mice were subsequently acquired on a clinical 3.0-T MR scanner. The LNs showed signal enhancement at all groups 30 min after injection, with more pronounced brightening effects at doses of 750 and 500 μg Mn/kg BW. Meanwhile, the color of LNs changed from green to blue on the *T*_1_-mapping pseudo-color images, implying shortening of relaxation time, indicating the accumulation of contrast agent in LNs. Figure 5B presents quantified the signal intensity changes caused by different dosages. Among them, data for 1000 μg Mn/kg BW were reproduced from Fig. 4B. The results showed that the increase in signal intensity remained significant at dosage as low as 125 μg Mn/kg BW (*P* < 0.05). The reduction in relaxation time also reflected the accumulation of MnC-20 in the LNs (Fig. 5C). Notably, possibly due to the high dosages of 750 and 500 μg Mn/kg BW, signal enhancement was also observed in the subcutaneous tissue of the thigh after administration, suggesting that diffusion of the contrast agent may occur in the intercellular matrix diffusion. Therefore, a dosage of 125 μg Mn/kg will be used for imaging of the SLN metastasis model.

### *In vivo* MR study of lymph nodes in metastatic mice

**Figure 6.**
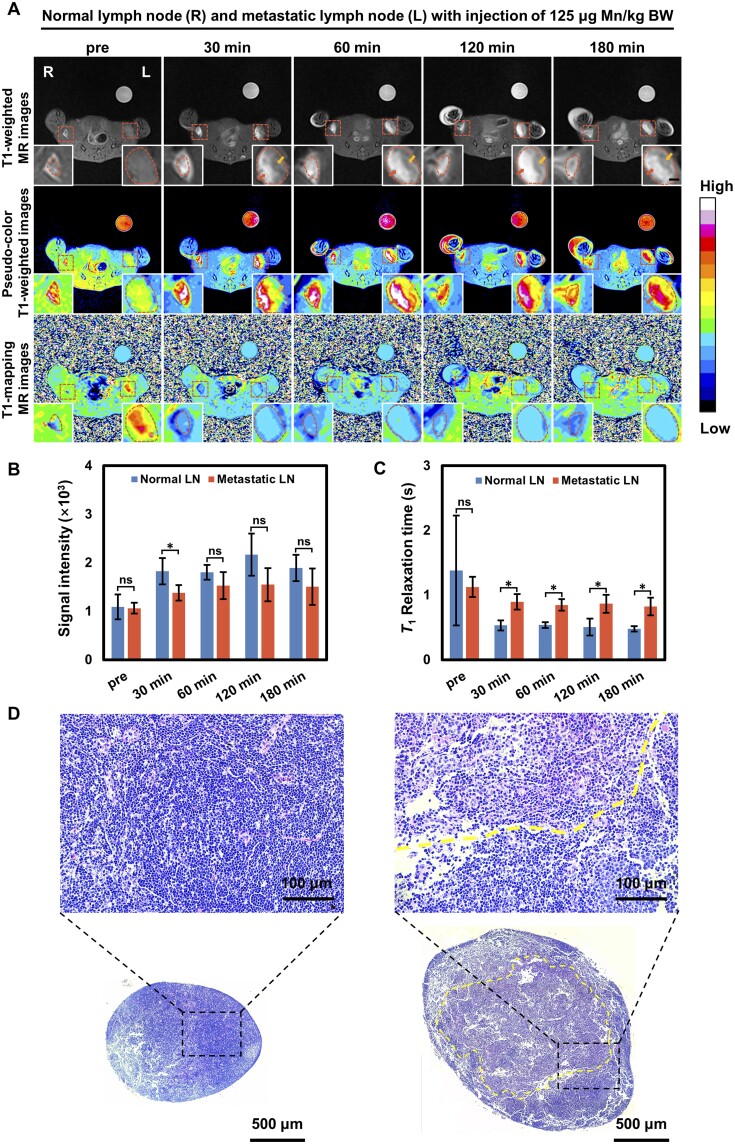
(**A**) Representative *T*_1_-weighted MR images, *T*_1_-weighted pseudo-color images and *T*_1_-mapping pseudo-color images of normal popliteal lymph nodes (L) and metastatic sentinel lymph nodes (R) before and after injection subcutaneously of MnC-20 at the dosage of 125 μg/kg BW (scalar bar = 1 mm); quantitative analysis of the signal intensity from *T*_1_-weighted MR images (**B**) and *T*_1_ relaxation time from *T*_1_-mapping images (**C**) (*n* = 4, *P < 0.05; **P < 0.01). (**D**) H&E staining images of normal popliteal lymph nodes and metastatic sentinel lymph nodes: area inside the red line contains tumor cells.

LN metastasis models were established using 4T1 breast cancer cells, in which the mice had tumor metastatic LNs on the left side and the normal ones on the right side. As shown in Fig. 6A, LNs in both sides were presenting low signal intensities in the *T*_1_-weighted images before injection. From 30 to 180 min after injection, signal enhancement can be observed in both normal LNs and tumor metastatic LNs, in which the normal LNs had relatively homogeneous brightness, but uneven signal intensity enhancement could be recognized in the tumor metastatic SLNs, with one part darker (yellow arrow) and the other brighter (red arrow). Because the metastatic LNs were occupied by tumor cells causing the uneven distribution of macrophages and less uptake of MnC-20, while the sufficient macrophages in normal LN lead the relatively homogeneous brightness [2, 4, 5, 27]. Therefore, uneven signal changes may be indicative of an occupying LN lesion and need to be further judged by quantitative analysis.

Quantitative analysis of mean signal intensity on *T*_1_-weighted images showed a significant difference (*P* < 0.05) between the metastasis LNs and the normal ones at 30 min (Fig. 6B). Although there was no statistical difference in terms of mean signal intensity after 60 min, the *T*_1_ relaxation time of normal LNs was much shorter than that of the metastatic SLNs and represented significantly different (*P* < 0.05). Hence, the higher *T*_1_ relaxation time could be the important indicate reflecting the tumor metastasis in LNs.

LNs were further analyzed by H&E staining after imaging studies. The LNs on the normal side had a uniform morphology with deep-stained nuclei, whereas larger tumor cell clusters with abnormal nuclei were observed in the LNs with the tumor metastasis, demonstrating the successful establishment of SLN metastasis models (Fig. 6D).

In short, MnC-20 at a dosage of 125 μg Mn/kg, administered for 30 min, could locate the suspected LNs by the inhomogeneity of signal intensity on the *T*_1_-weighted images, and the average value of signal intensity was lower than that of normal ones, which was favorable in distinguishing SLNs from the normal ones.

## Conclusion

In this study, a series of manganese-based hybrid MnCs were constructed using PEG monooctadecyl ether with different PEG molecular weights. All of the manganese complexes have small particle size and can enter the draining LNs rapidly. The nanoparticles constructed by C18E10, C18E20 and C18E50 showed slight differences in particle size and relaxivity, and the slight increase in PEG molecular weight contributed to the increase of size and the enhancement of relaxivity. The modified PhDTA ligands in the micelles were still having a high kinetic inertness similar to PhDTA themselves. In addition, the *in vivo* contrast enhancement ability in LNs of the MnCs was evaluated using 4T1 tumor metastasis mice models. The results showed that at a dosage of 125 μg Mn/kg, a significant discrimination between metastatic and normal LNs was achieved 30 min after administration. This study serves as a useful reference for design of manganese-based nanoprobes for LN imaging.

## Supplementary Material

rbad053_Supplementary_Data
