# Supplementary material for: Kinetically inert manganese (II)-based hybrid micellar complexes for magnetic resonance imaging of lymph node metastasis
Source: Regen Biomater. 2023 May 25;10:rbad053. doi: 10.1093/rb/rbad053 (PMC10244211; doi:10.1093/rb/rbad053)
Supplement: rbad053_Supplementary_Data [file rbad053_supplementary_data.docx]

**Supporting Information**

**Kinetically inert manganese (II) -based hybrid micellar complexes for magnetic resonance imaging of lymph node metastasis**

Kai Chen^1 #^, Zhongyuan Cai^1 #^, Yingzi Cao^1^, Lingling Jiang^1^, Yuting Jiang^1^, Haojie Gu^1^, Shengxiang Fu^1^, Chunchao Xia^2^, Su Lui^2^, Qiyong Gong^3,4^, Bin Song^2^, Hua Ai^1, 2*^

1. National Engineering Research Center for Biomaterials, Sichuan University, Chengdu 610064, China
2. Department of Radiology, West China Hospital, Sichuan University, Chengdu 610041, China
3. Huaxi MR Research Center (HMRRC), West China Hospital of Sichuan University, Chengdu, China
4. Psychoradiology Research Unit of Chinese Academy of Medical Sciences, Sichuan University, Chengdu, China

* Corresponding author:

Hua Ai, National Engineering Research Center for Biomaterials, Sichuan University, Chengdu 610064, P. R. China. Phone: 86-28-85413991, Email: huaai@scu.edu.cn


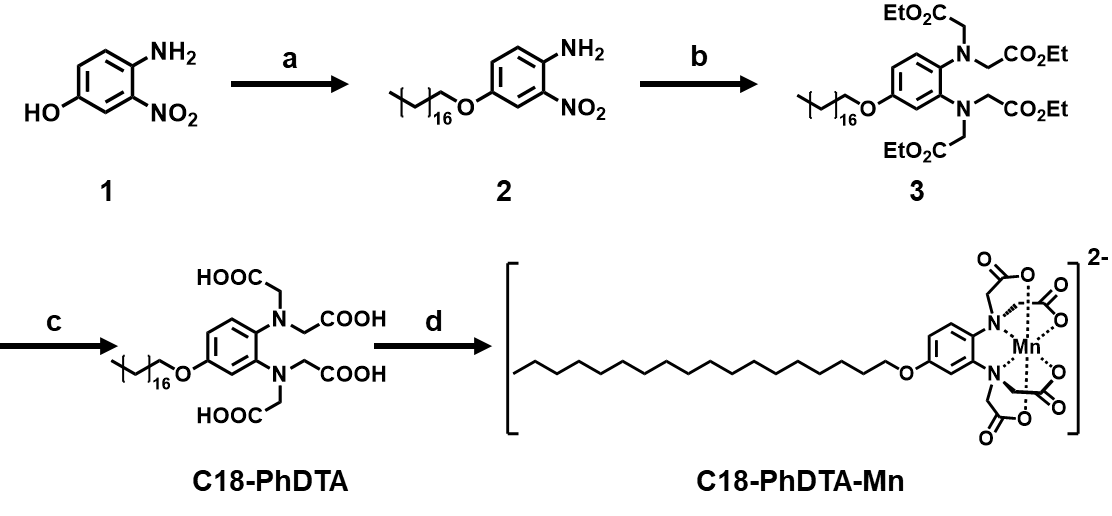


**Scheme S1.** The synthetic route of the C18-PhDTA-Mn.


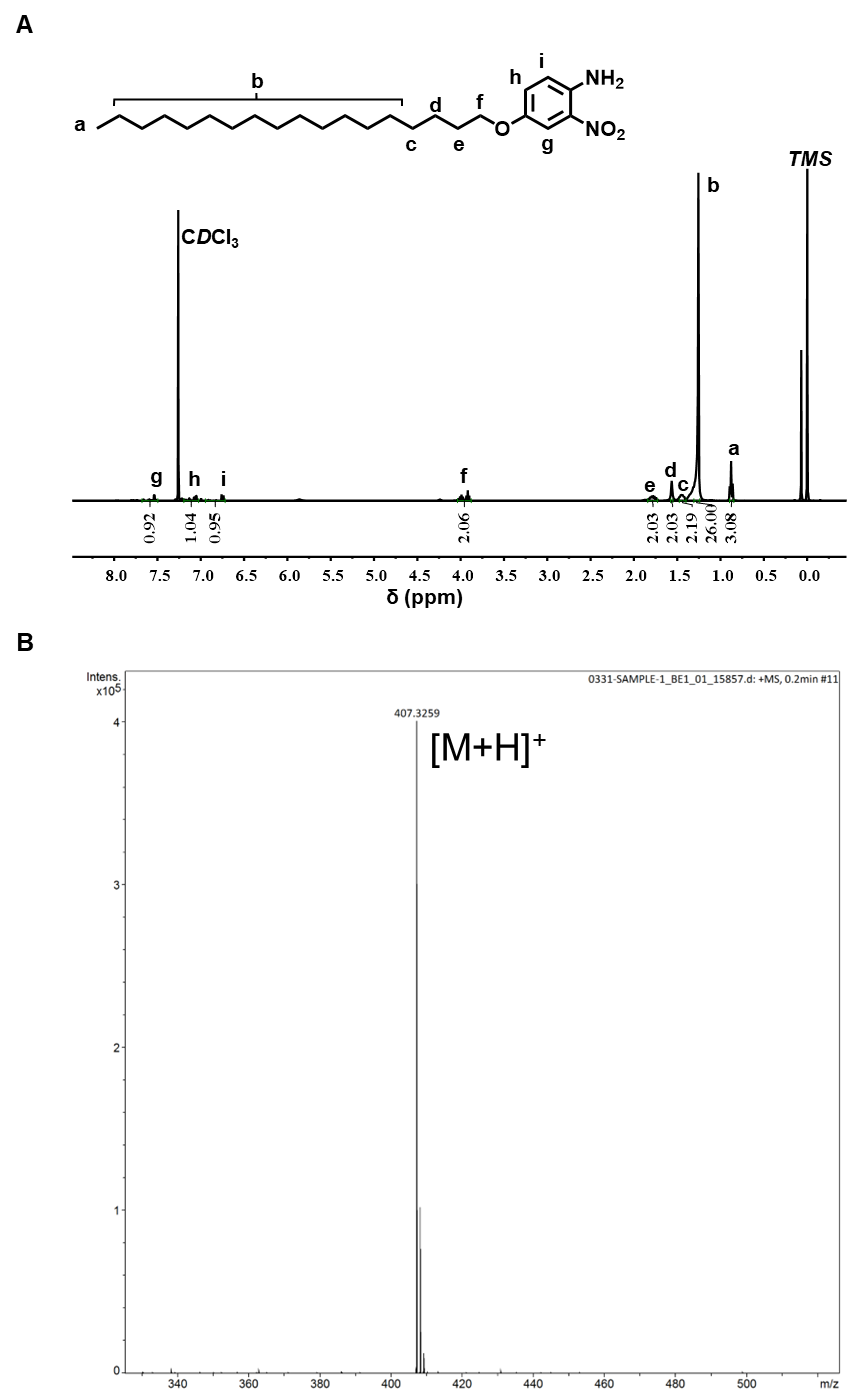


**Figure S1.** ^1^H NMR spectra (A) (400 MHz, CDCl_3_) and electrospray ionization mass spectrometry (ESI-MS) spectrum (B) of compound 2.


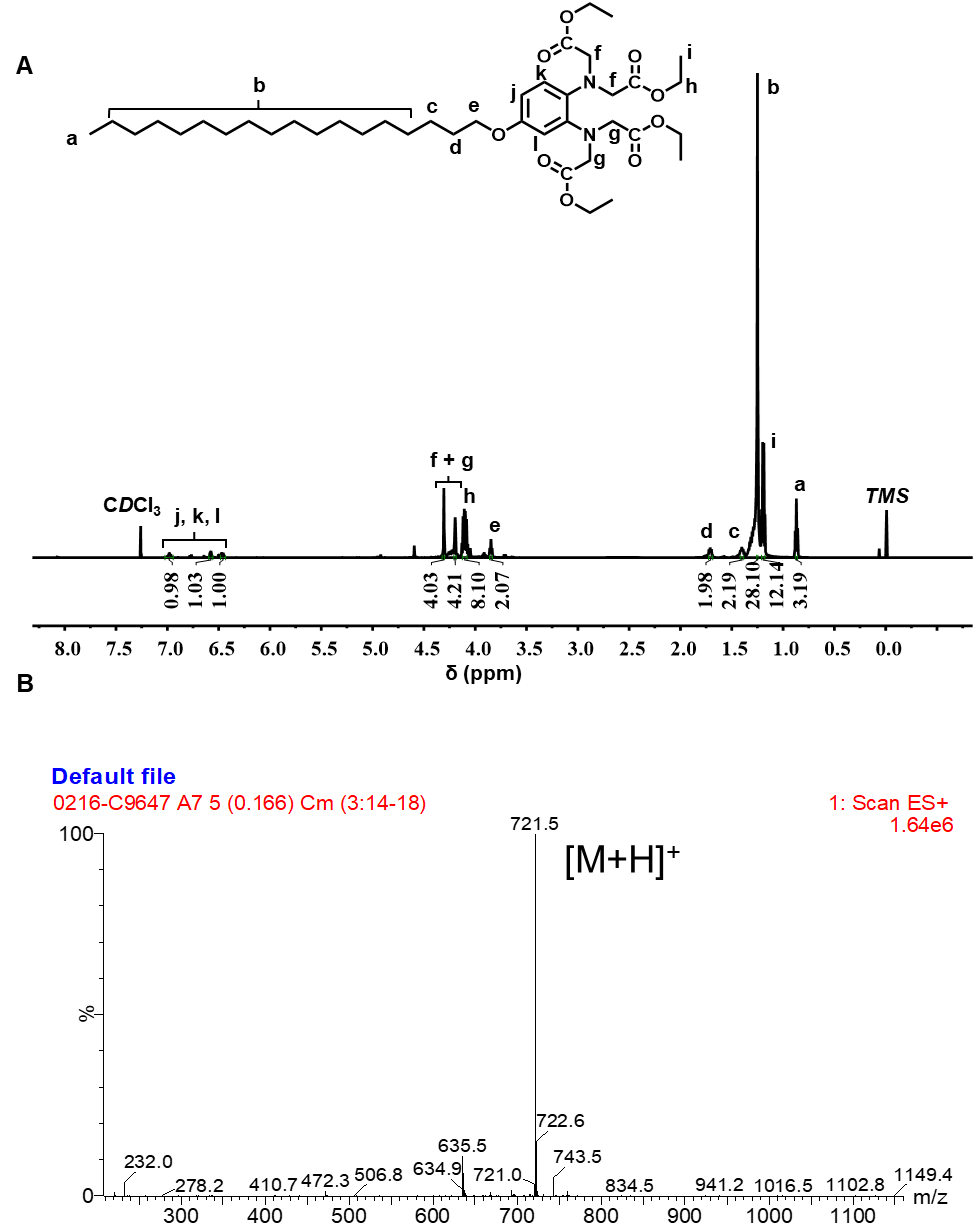


**Figure S2.** ^1^H NMR spectra (A) (600 MHz, CDCl_3_) and ESI-MS spectrum (B) of compound 3.


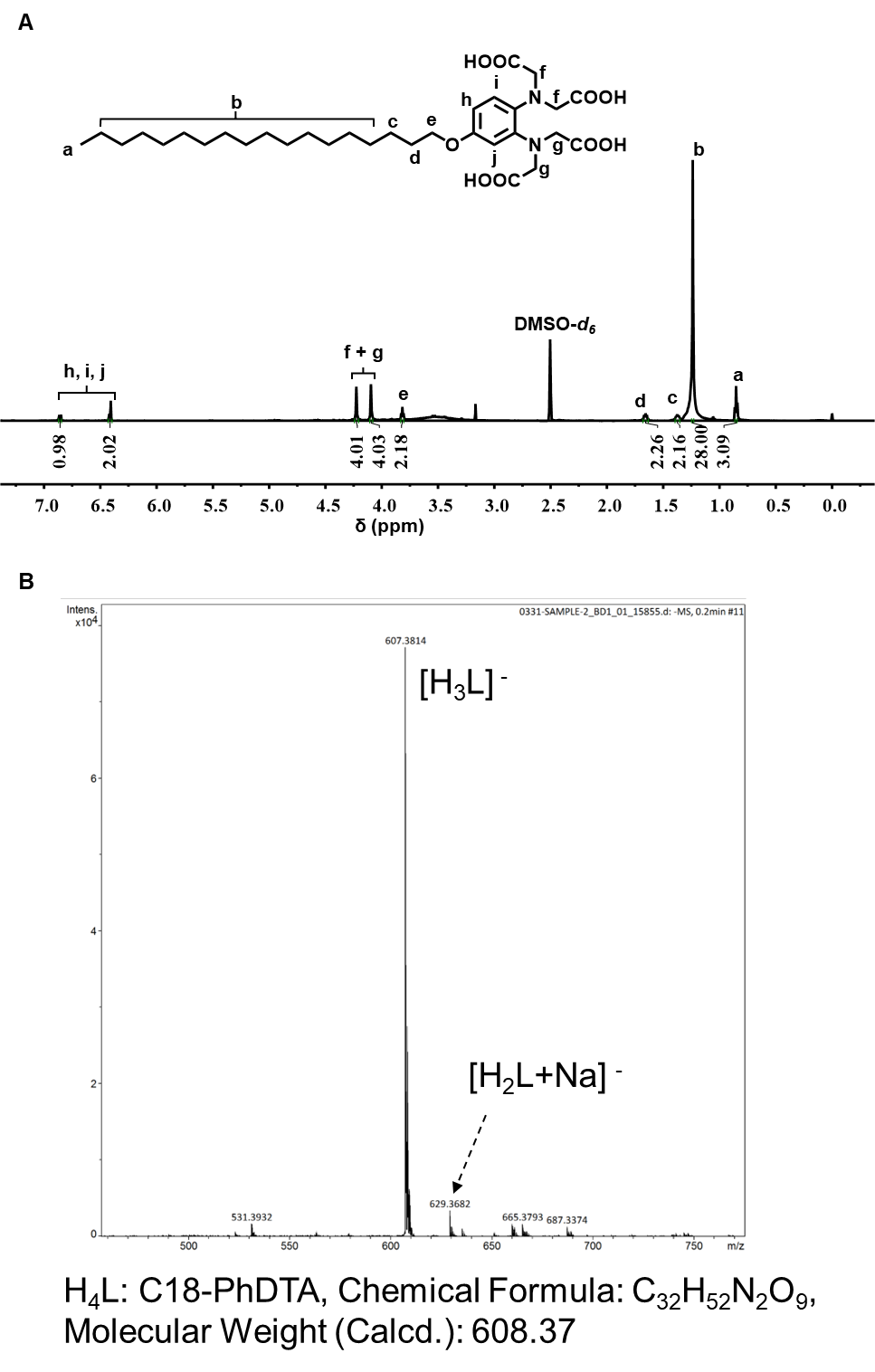


**Figure S3.** ^1^H NMR spectra (A) (600 MHz, DMSO-*d*_6_) and ESI-MS spectrum (B) of C18-PhDTA.


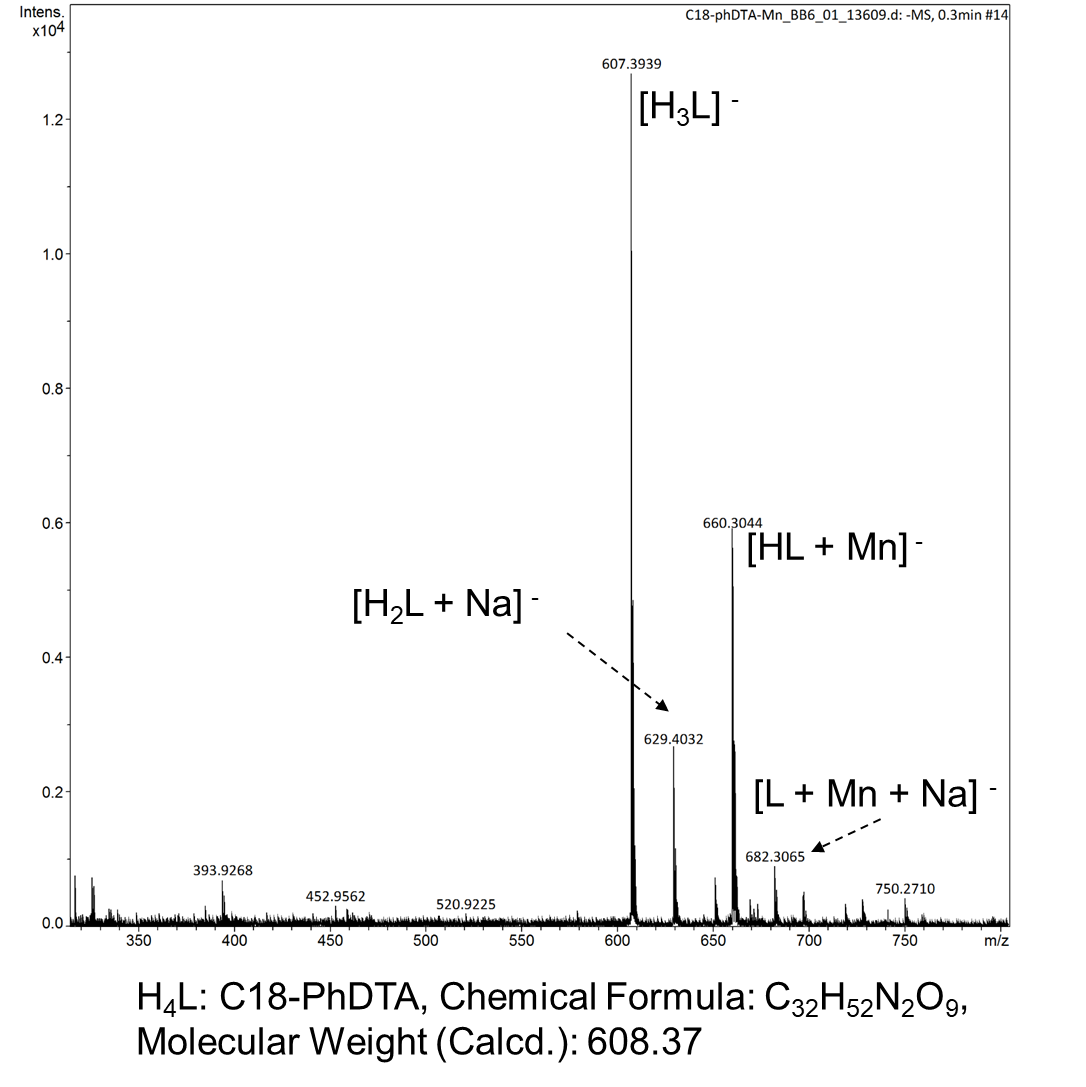


**Figure S4.** ESI-MS spectrum of C18-PhDTA-Mn.


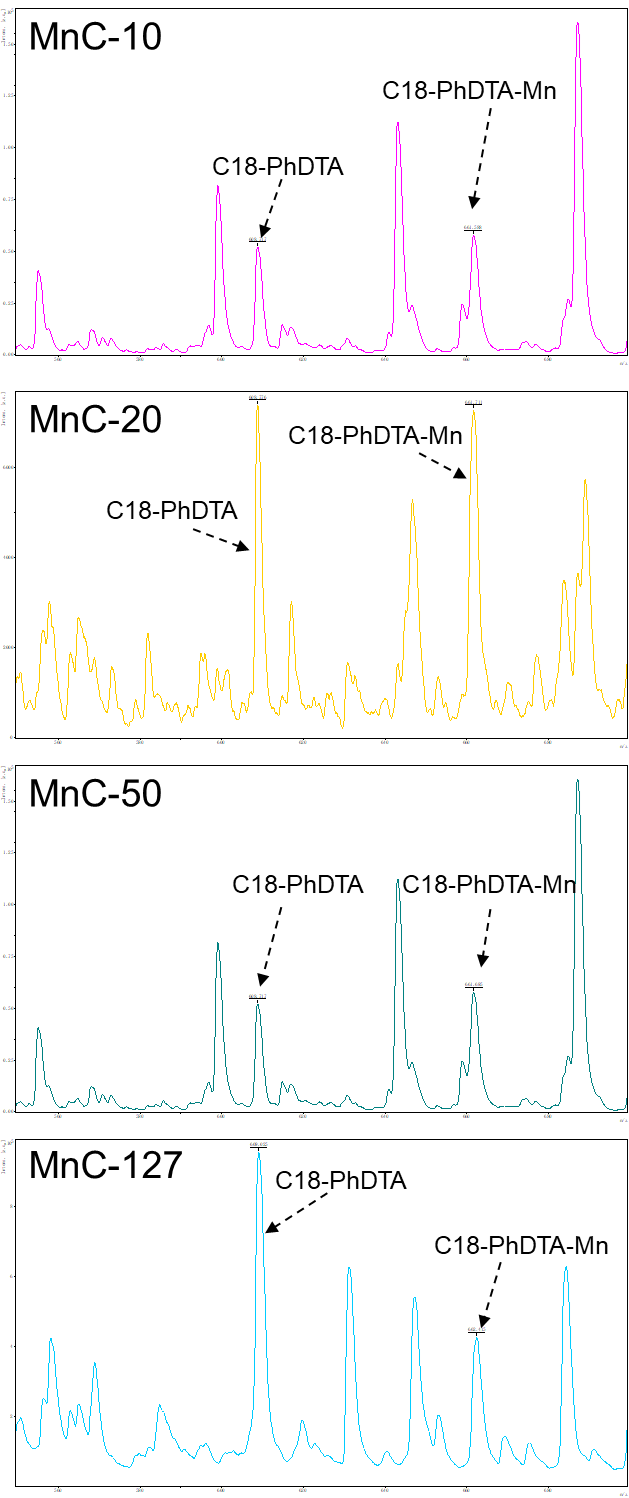


**Figure S5.** Matrix-assisted laser desorption ionization time of flight (MALDI-TOF) mass spectrometry of MnC-10, MnC-20, MnC-50 and MnC-127.


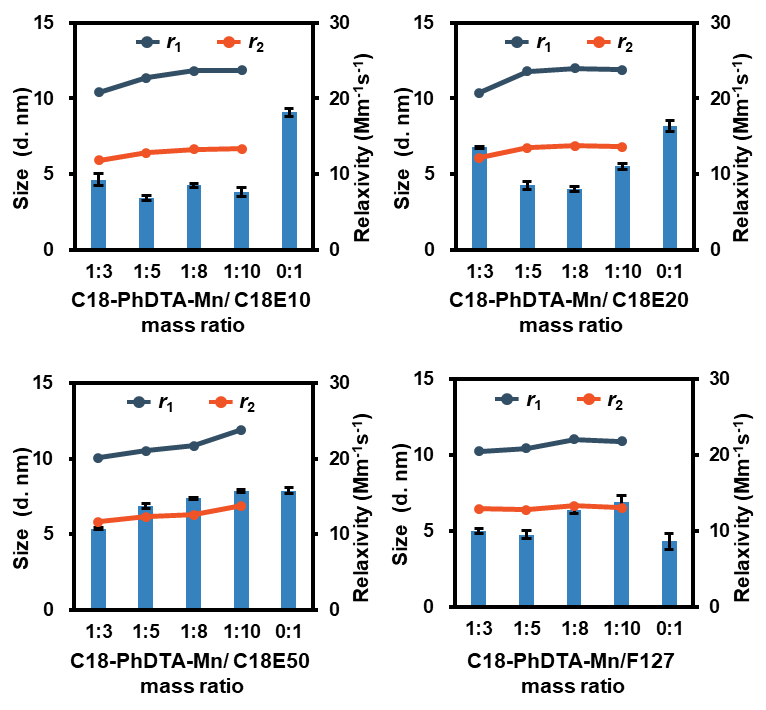


**Figure S6.** The hydrodynamic particle size and relaxivities at 1.5 T of manganese-based hybrid micelles prepared with different mass ratios (1:3, 1:5, 1:8 and 1:10) of C18-PhDTA/PEG-based amphiphilic polymer (C18E10, C18E20, C18E50 and F127).


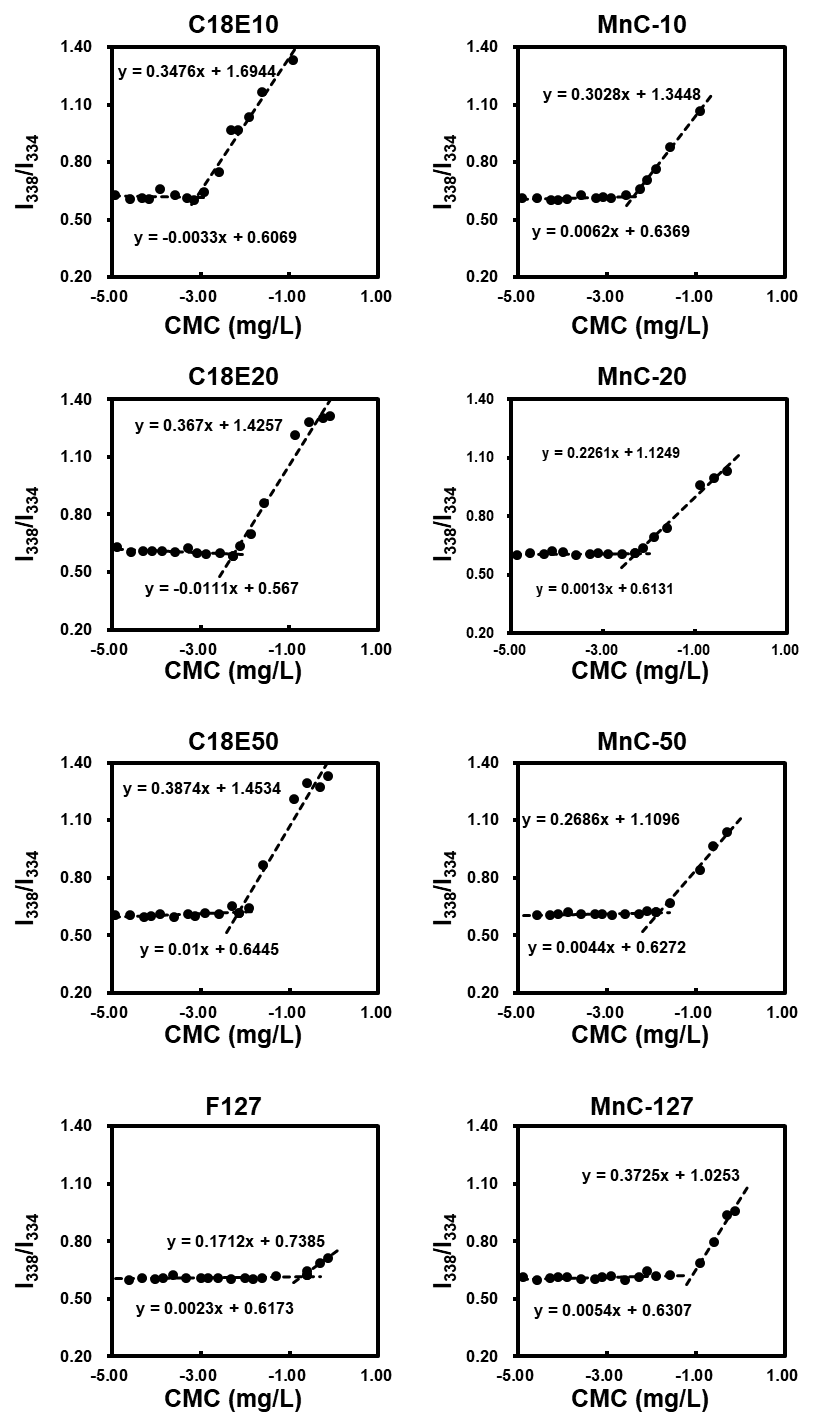


**Figure S7.** Determination curves of critical micelle concentration (CMC) of MnC-10, MnC-20, MnC-50, MnC-127, and PEG-based polymers (C18E10, C18E20, C18E50, and F127).


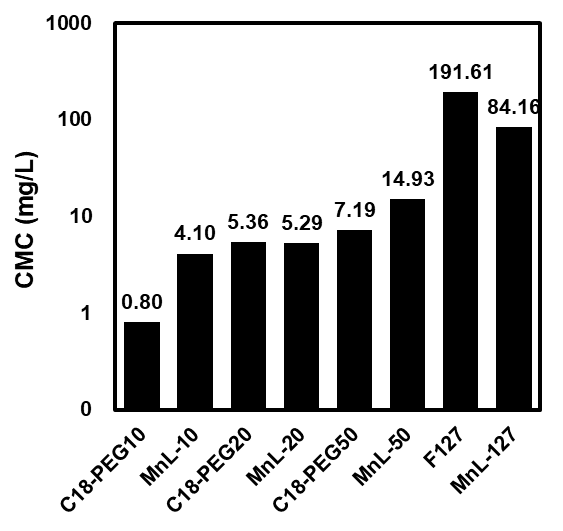


**Figure S8.** CMC of MnC-10, MnC-20, MnC-50, MnC-127, and PEG-based polymers (C18E10, C18E20, C18E50, and F127).


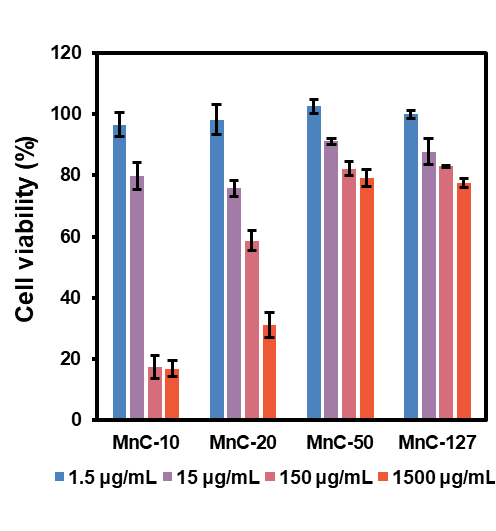


**Figure S9.** Cell viabilities of Raw264.7 incubated with different concentrations of MnC-10, MnC-20, MnC-50 and MnC-127 for 12 h.


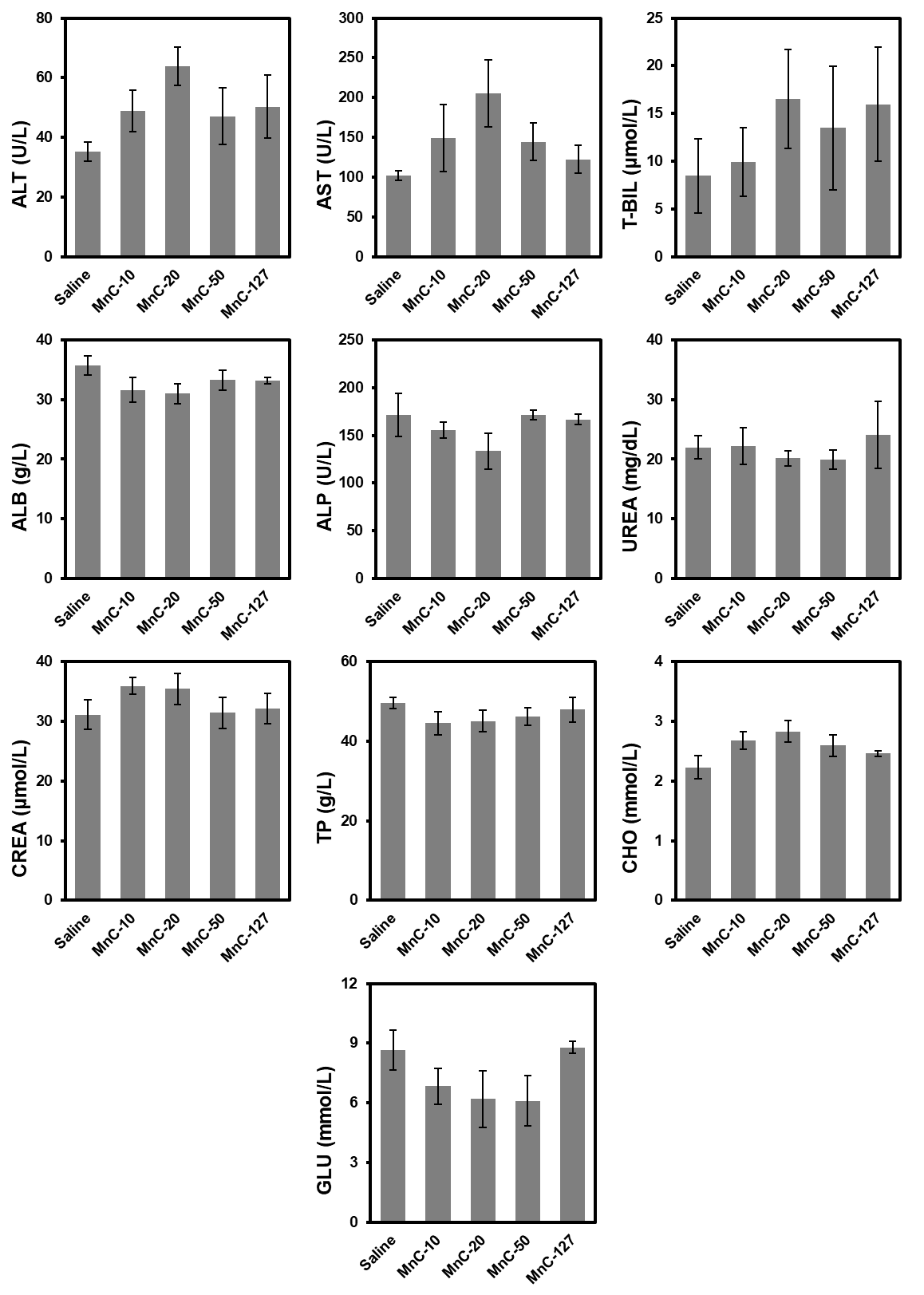


**Figure S10.** Serum biochemical indicators of mice 24 h after administration of MnC-10, MnC-20, MnC-50, MnC-127 and saline, including alanine transferase (ALT, reference range: 10.06-96.47), aspartate transferase (AST, reference range: 36.31-235.48), total bilirubin (T-BIL, reference range: 6.09-53.06), albumin (ALB, reference range: 21.22-39.15), alkaline phosphatase (ALP, reference range: 22.52-474.35), the blood urea nitrogen (UREA, reference range: 10.81-34.74), creatinine (CREA, reference range: 10.91-85.09), Serum total protein (TP, reference range: 38.02-75.06), Cholesterol (CHO, reference range: 2.05-4.16), the blood glucose (GLU, reference range: 4.66-13.42).


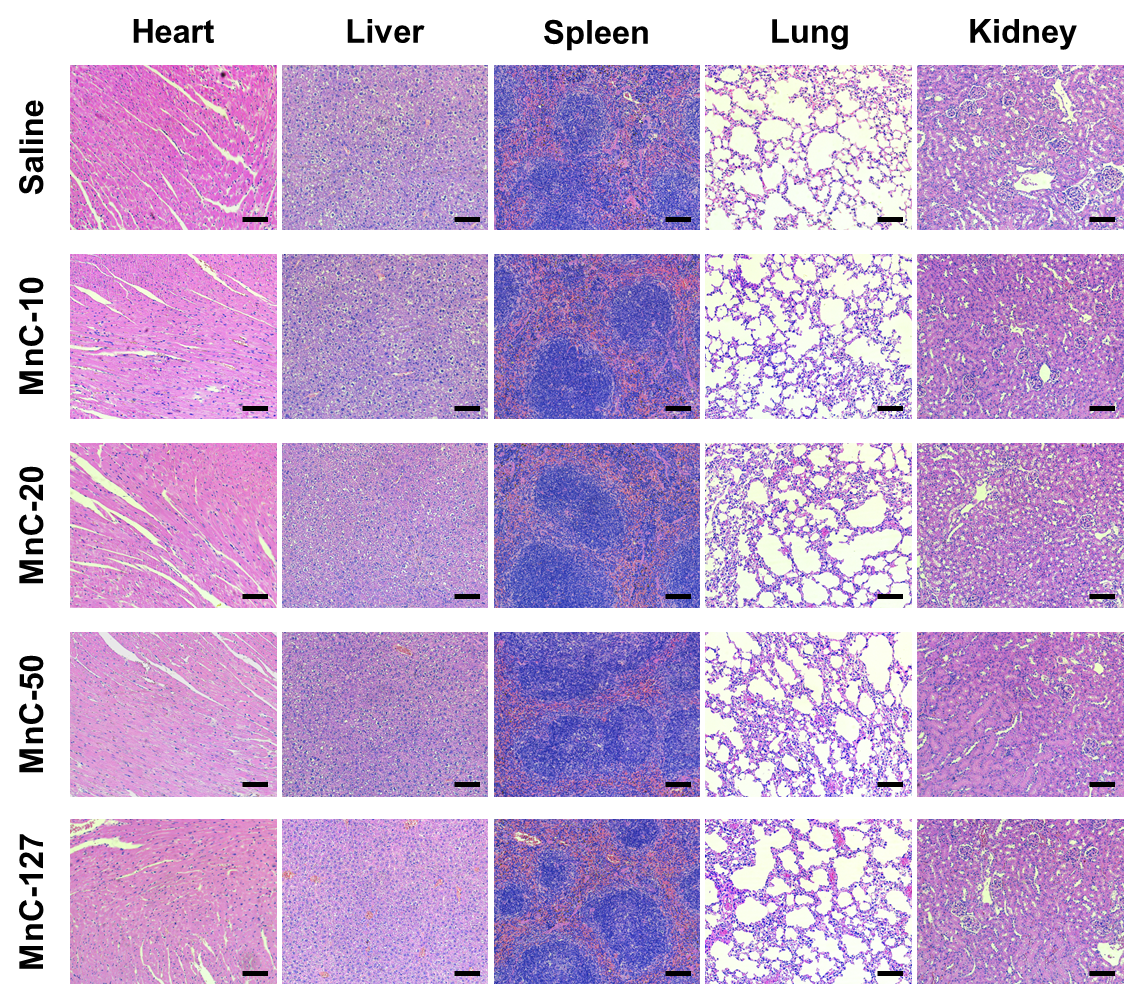


**Figure S11.** H&E staining of main issues from mice 24 h after administration of MnC-10, MnC-20, MnC-50, MnC-127 and saline. (Scale bar = 100 μm)


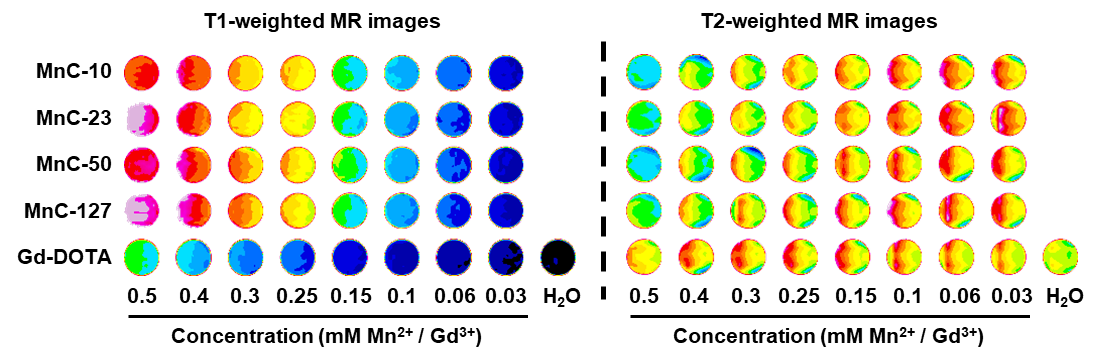


**Figure S12.** Pseudo-color T1-weighted MR images at 1.5 T corresponding to Figure 3.


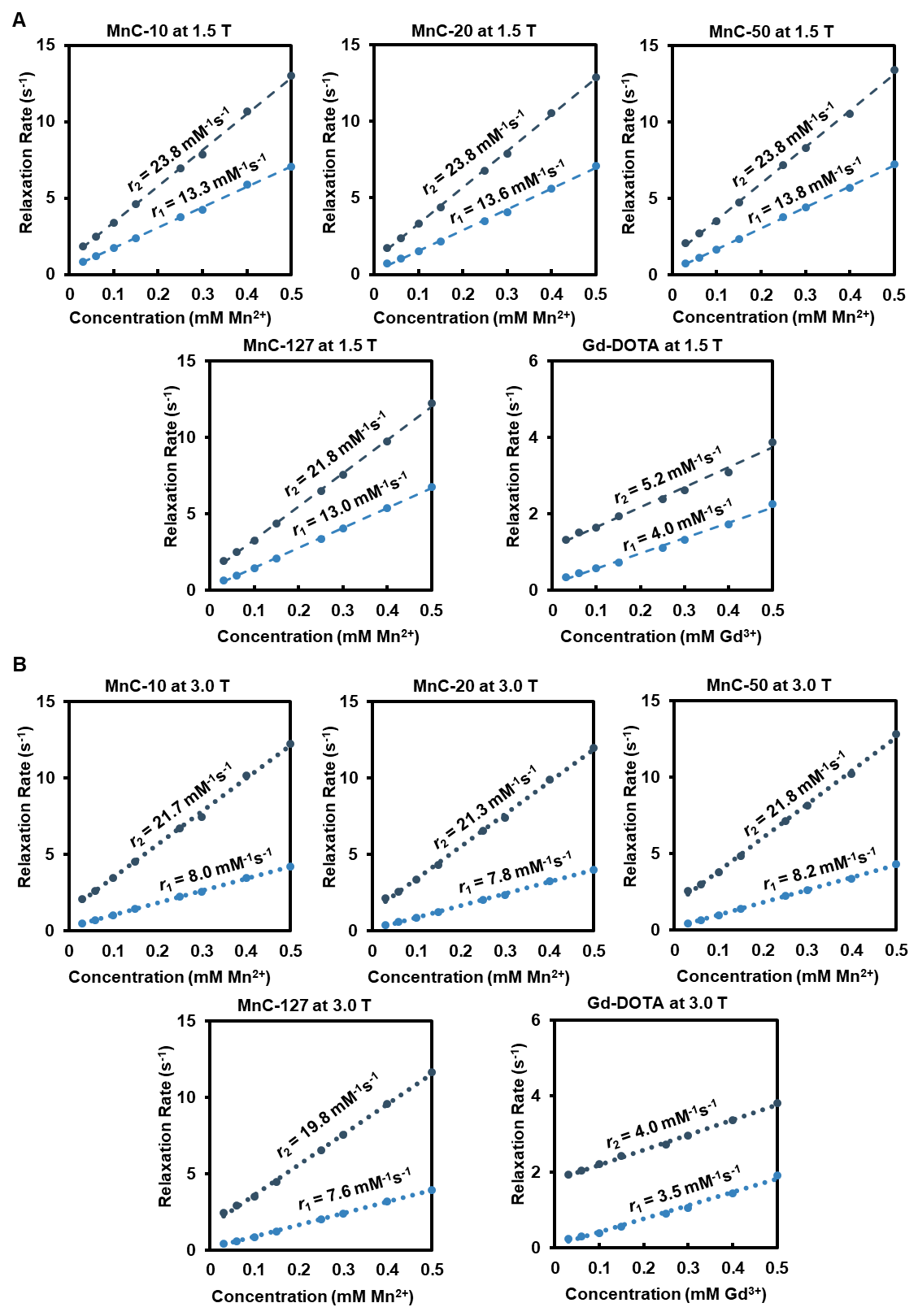


**Figure S13.** Longitudinal relaxivity (*r*_1_) and transverse relaxivity (*r*_2_) of MnC-10, MnC-20, MnC-50, MnC-127 and Gd-DOTA at 1.5 T (A) and 3.0 T (B), respectively.

**Table S1.** The hydrodynamic particle size of manganese-based hybrid micelles prepared with different mass ratios (0:1, 1:1, 1:3, 1:5, 1:8 and 1:10) of C18-PhDTA/PEG-based amphiphilic polymer (C18E10, C18E20, C18E50 and F127).

| **Size (d. nm)** | | **Mass ratios of C18-PhDTA-Mn/polymer** | | | | | |
| --- | --- | --- | --- | --- | --- | --- | --- |
|  |  | **1:1** | **1:3** | **1:5** | **1:8** | **1:10** | **0:1** |
| **Class of amphiphilic polymer** | **C18E10** | 360.5 | 4.6 | 3.4 | 4.2 | 3.8 | 9.1 |
|  | **C18E20** | 7.4 | 6.8 | 4.2 | 4.0 | 5.5 | 8.2 |
|  | **C18E50** | 379.5 | 5.4 | 6.9 | 7.4 | 7.9 | 7.9 |
|  | **F127** | 920.1 | 5.0 | 4.7 | 6.4 | 6.9 | 4.3 |

**Table S2.** Longitudinal relaxivities at 1.5 T of manganese-based hybrid micelles prepared with different mass ratios (1:3, 1:5, 1:8 and 1:10) of C18-PhDTA/PEG-based amphiphilic polymer (C18E10, C18E20, C18E50 and F127).

| ***r*_1_ (mM^-1^s^-1^) at 1.5 T** | | **Mass ratios of C18-PhDTA-Mn/polymer** | | | |
| --- | --- | --- | --- | --- | --- |
|  |  | **1:3** | **1:5** | **1:8** | **1:10** |
| **Class of amphiphilic polymer** | **C18E10** | 10.0 | 11.8 | 12.8 | 13. 3 |
|  | **C18E20** | 11.7 | 12.1 | 13.5 | 13.8 |
|  | **C18E50** | 11.7 | 12.3 | 12.6 | 13.8 |
|  | **F127** | 12.9 | 12.8 | 13.3 | 13.0 |

**Table S3.** Transverse relaxivities at 1.5 T of manganese-based hybrid micelles prepared with different mass ratios (1:3, 1:5, 1:8 and 1:10) of C18-PhDTA/PEG-based amphiphilic polymer (C18E10, C18E20, C18E50 and F127).

| ***r*_2_ (mM^-1^s^-1^) at 1.5 T** | | **Mass ratios of C18-PhDTA-Mn/polymer** | | | |
| --- | --- | --- | --- | --- | --- |
|  |  | **1:3** | **1:5** | **1:8** | **1:10** |
| **Class of amphiphilic polymer** | **C18E10** | 20.9 | 22.7 | 23.7 | 23.8 |
|  | **C18E20** | 20.8 | 23.6 | 24.0 | 23.8 |
|  | **C18E50** | 20.2 | 21.1 | 21.7 | 23.8 |
|  | **F127** | 20.5 | 20.9 | 22.0 | 21.8 |

**Table S4.** Longitudinal relaxivities at 3.0 T of manganese-based hybrid micelles prepared with different mass ratios (1:3, 1:5, 1:8 and 1:10) of C18-PhDTA/PEG-based amphiphilic polymer (C18E10, C18E20, C18E50 and F127).

| ***r*_1_ (mM^-1^s^-1^) at 3.0 T** | | **Mass ratios of C18-PhDTA-Mn/polymer** | | | |
| --- | --- | --- | --- | --- | --- |
|  |  | **1:3** | **1:5** | **1:8** | **1:10** |
| **Class of amphiphilic polymer** | **C18E10** | 7.3 | 7.9 | 8.1 | 8.0 |
|  | **C18E20** | 7.3 | 7.8 | 7.9 | 7.8 |
|  | **C18E50** | 7.3 | 7.6 | 7.7 | 8.2 |
|  | **F127** | 7.9 | 7.6 | 7.9 | 7.6 |

**Table S5.** Transverse relaxivities at 3.0 T of manganese-based hybrid micelles prepared with different mass ratios (1:3, 1:5, 1:8 and 1:10) of C18-PhDTA/PEG-based amphiphilic polymer (C18E10, C18E20, C18E50 and F127).

| ***r*_2_ (mM^-1^s^-1^) at 3.0 T** | | **Mass ratios of C18-PhDTA-Mn/polymer** | | | |
| --- | --- | --- | --- | --- | --- |
|  |  | **1:3** | **1:5** | **1:8** | **1:10** |
| **Class of amphiphilic polymer** | **C18E10** | 19.0 | 21.0 | 22.1 | 21.7 |
|  | **C18E20** | 18.8 | 21.5 | 21.7 | 21.3 |
|  | **C18E50** | 18.0 | 19.0 | 19.9 | 21.8 |
|  | **F127** | 18.4 | 18.8 | 20.2 | 19.8 |
